# Population-Level Effectiveness of COVID-19 Vaccination Program in the United States: Causal Analysis Based on Structural Nested Mean Model

**DOI:** 10.3390/vaccines10050726

**Published:** 2022-05-05

**Authors:** Rui Wang, Jiahao Wang, Taojun Hu, Xiao-Hua Zhou

**Affiliations:** 1 Department of Biostatistics, School of Public Health, Peking University, Beijing 100191, China; wangruiruishou@pku.edu.cn (R.W.); 2011110158@stu.pku.edu.cn (T.H.); 2 School of Public Health, Peking University, Beijing 100191, China; jiahaowang@pku.edu.cn; 3 China Center for Health Development Studies, Peking University, Beijing 100191, China; 4 Beijing International Center for Mathematical Research, Peking University, Beijing 100871, China

**Keywords:** COVID-19, vaccines, causal inference, effectiveness, structural nested mean models

## Abstract

Though COVID-19 vaccines have shown high efficacy, real-world effectiveness at the population level remains unclear. Based on the longitudinal data on vaccination coverage and daily infection cases from fifty states in the United States from March to May 2021, causal analyses were conducted using structural nested mean models to estimate the population-level effectiveness of the COVID-19 vaccination program against infection with the original strain. We found that in the US, every 1% increase of vaccination coverage rate reduced the weekly growth rate of COVID-19 confirmed cases by 1.02% (95% CI: 0.26%, 1.69%), and the estimated population-level effectiveness of the COVID-19 program was 63.9% (95% CI: 18.0%, 87.5%). In comparison to a no-vaccination scenario, the COVID-19 vaccination campaign averted 8.05 million infections through the study period. Scenario analyses show that a vaccination program with doubled vaccination speed or with more rapid vaccination speed at the early stages of the campaign would avert more infections and increase vaccine effectiveness. The COVID-19 vaccination program demonstrated a high population-level effectiveness and significantly reduced the disease burden in the US. Accelerating vaccine rollout, especially at an early stage of the campaign, is crucial for reducing COVID-19 infections.

## 1. Introduction

Since December 2019, the coronavirus disease 2019 (COVID-19) pandemic has rapidly spread and caused severe disease burden globally [1]. Countries worldwide have strived to boost COVID-19 vaccine coverage to contain the spread of the virus and reduce the burden of severe disease [2]. In the United States (US), three COVID-19 vaccines, developed by Pfizer-BioNTech, Moderna, and Johnson & Johnson, were distributed for public use, and the coverage rate of at least one dose of the COVID-19 vaccine rapidly increased from 1.3% on 1 January 2021 to 64.0% on 1 October 2021 among the general population of the United States [3].

Despite randomized clinical trials showing high efficacy of COVID-19 vaccines against infection (ranging from 66.9–95%) [4,5,6], the real-world effectiveness of the vaccines at the population level remains unclear. The vaccine direct effectiveness, which is a measure of the direct effect of the vaccine on vaccinated individuals, has been evaluated at the individual level using cohort studies (from the same population) [7,8,9,10] or case-control studies [11], and their results showed a lower risk of infection among vaccinated individuals compared with those unvaccinated. However, these designs are limited in capturing herd immunity (indirect effect) and population-level effectiveness (overall effect of a vaccination program in the population) [12,13,14]. Furthermore, these designs were unable to examine the population-level outcomes of mass vaccination (e.g., the growth rate of infection cases [15,16,17,18], the number of cases averted [19]), which have important public health implications and provide evidence for policymaking. Some methods have been adopted to study the population-level effect (overall effect) of the COVID-19 vaccines, most of which are mathematical models. For example, compartment models and agent-based models are built to simulate how cases could evolve under different COVID-19 vaccination strategies [20,21,22,23,24]. However, these models rely on estimated parameters unconnected to the actual epidemic and vaccination data.

The challenges in evaluating the population-level effectiveness of the COVID-19 vaccines are the utilization and integration of real-world data on the epidemic and vaccination that evolved dramatically over time. The relationship between epidemic outcome (e.g., growth rate of new cases) and vaccinated population is complex and dynamic because various factors influenced vaccine uptake, such as perceptions of vaccination [25], severity of the epidemic, and intensity of non-pharmaceutical interventions [26,27]. The time-varying characteristics of these factors may confound the relationship between the vaccinated population at the present stage and the growth rate of infection cases in the subsequent period, and they may also be impacted by the vaccinated population (treatment) and the growth rate of new infection cases (outcome) at the previous stage. In the past, there were studies that used longitudinal data at the population level as statistical models to assess the effectiveness of other vaccines, such as influenza vaccine, meningococcal B vaccine, and BCG vaccine. However, no such studies were conducted regarding COVID-19 vaccination, to the best of our knowledge. Furthermore, in the context of COVID-19 vaccination, these methods are unable to handle the rapidly evolving relationship between time-varying treatments, outcomes, and confounders. For example, generalized estimating equations [28] or fixed-effects models [29] will generate biased estimates when there exist time-varying confounders that are impacted by previous independent variables [30,31,32,33]. The Suspectible-Infected-Recovered (SIR) model cannot handle confounders, either. As a result, these methods only give predictions based on the associations in observed data and lack causal interpretation due to the failure to adjust for confounders. Synthetic control [34] or interrupted time series [35] can only evaluate binary treatment.

In this paper, we studied the relationship between the vaccinated population and epidemic outcomes at population level. Structural nested mean models (SNMMs) were used to deal with time-varying confounders [36]. SNMMs can easily handle continuous treatments [36,37,38]. Moreover, as defined in the potential outcome framework [39,40], SNMMs have the advantage of providing a causal interpretation of the estimates and results. Based on the longitudinal data on COVID-19 vaccination coverage and weekly confirmed infection cases in the United States, we estimated the effect of the vaccinated population on the growth rate of confirmed new cases using SNMMs. We then calculated the population-level effectiveness of the COVID-19 vaccination program against the original COVID-19 virus strain and the disease burden averted during the study period, compared with a no-vaccination scenario. This study identified that every 1% increase in vaccine coverage would reduce the weekly growth rate of COVID-19 cases by 1.02% (95% CI: 0.26%, 1.69%), and stressed the significance of intensive vaccine rollout, especially at early stages, in averting infections and increasing vaccine effectiveness.

## 2. Materials and Methods

### 2.1. Data

We collected state-level daily COVID-19 cases and vaccine coverage data from 1 March to 30 May 2021 in the U.S. from Johns Hopkins Coronavirus Resource Center and the Centers for Disease Control and Prevention (CDC) [1,3]. We also collected state baseline characteristics, including GDP, population, health resource (number of physicians), political position (red or blue states in the 2016 election), proportion of people aged 65 or above, race composition, education level (proportion of people with advanced degrees), unemployment rate, and sex ratio. Their sources can be found in Appendix A Table A1. Overall government response index was collected from the Oxford COVID-19 Government Response Tracker dataset [41].

Data from 49 states and Washington, DC, in the U.S. were included in the analyses, excluding New Hampshire due to errors in the vaccinated population data. The study aimed to evaluate the population-level effectiveness of COVID-19 vaccination program against the original strain. Therefore, the period after June 2021 was excluded because the proportion of SARS-CoV-2 variants changed radically since then, making the Delta variant gradually predominate as the main variant of transmission. The periods before March 2021 were also excluded due to missing data on vaccination coverage. The data were aggregated at the weekly level to avoid the collection and report patterns of the confirmed case data, resulting in the study period of 13 weeks at 50 states (including DC) for analysis (first week: 1 March to 7 March; 13th week: 24 May to 30 May).

### 2.2. Treatment and Outcome Variable

Treatment (denoted as $A_{i,t}$) represents the number of people (per 10,000) who received their first dose in state i at week t. The key outcome variable, the growth rate of new COVID-19 cases for each week [15,16,17,18], is calculated as

(1) $$Y_{i,t}=log\left(C_{i,t+1}\right)-log\left(C_{i,t}\right),$$

where $C_{i,t}$ is the number of new cases reported in state i at week t, and $Y_{i,t}$ represents the growth rate of new cases in state i at week t. We omitted index i when there was no ambiguity.

### 2.3. Causal Parameters of Interest

To define our causal parameters of interest, we formalized our ideas in a potential outcome framework, which was proposed by Donald Rubin [40] and extended to longitudinal settings by James Robins [42]. For each state, our data are of the form $\left\{L_{1},Y_{1},A_{1},\ldots ,L_{13},Y_{13},A_{13},Y_{14}\right\}$, where $L_{t}$ denotes covariates, including time-varying covariates and baseline covariates (we mention which confounders we included in analysis in the next section). Assume at week t, first $L_{t}$ was observed, then $Y_{t}$ was observed, and $A_{t}$ was the last to be observed.

Let $X_{t}$ be an arbitrary variable. We use ${\bar{X}}_{t}$ to denote the history of $X_{t}$, that is to say, ${\bar{X}}_{t}=\left(X_{1},\ldots .,X_{t}\right)$. Then, ${\bar{A}}_{t}=\left(A_{1},\ldots ,A_{t}\right)$ represents the vaccinated population (per 10,000 people) each week prior to t; we refer ${\bar{a}}_{t}$ as vaccination speed because it represents the first-order difference in vaccination coverage each week. The potential outcome can be written as $Y_{t+1}\left({\bar{a}}_{t}\right)$, which is the outcome that would have been observed if the vaccination population had been set to ${\bar{A}}_{t}={\bar{a}}_{t}$. Moreover, we use $Y_{t+1}\left({\bar{a}}_{s},0\right)$ for abbreviation of $Y_{t+1}\left({\bar{a}}_{s},0,\ldots ,0\right)$, which is the potential outcome under intervention which takes the value ${\bar{a}}_{s}$ at the first s periods and takes value 0 for the remaining periods. The potential outcomes and observed outcomes are linked by the consistency assumption [33], which means that if a region’s observed vaccination speed at stage t+1 is ${\bar{a}}_{t}$, then $Y_{t+1}=Y_{t+1}\left({\bar{a}}_{t}\right)$.

Our first causal parameter of interest is the average treatment effect [31]:

(2) $$E\left[Y_{t+1}\left({\bar{a}}_{t}{}^{\left(1\right)}\right)-Y_{t+1}\left({\bar{a}}_{t}^{\left(2\right)}\right)\right].$$

This is the contrast of the mean difference of outcome under different vaccination speeds ${\bar{a}}_{t}{}^{\left(1\right)}$ and ${\bar{a}}_{t}{}^{\left(2\right)}$, representing the effect of vaccination speed (vaccine population per 10,000 people) before t+1 on the growth rate.

Moreover, how many new cases we would have observed each week in the United States if a certain vaccination speed ${\bar{a}}_{i,\;13}^{*}$ had been accomplished is also of interest to us, and is

(3) $$\overset{50}{\underset{i=1}{\sum }}C_{i,t+1}\left({\bar{a}}_{i,t}^{*}\right).$$

Here $C_{i,t+1}\left({\bar{a}}_{i,t}^{*}\right)$ is the potential outcome of new cases at state i and week t, and ${\bar{a}}_{i,t}^{*}$ is the segment of first t components of ${\bar{a}}_{i,\;13}^{*}$.

Lastly, define the population-level vaccine effectiveness (PVE) of vaccination speed ${\bar{a}}_{i,\;13}^{*}$ as

(4) $$1-\frac{\sum_{t=1}^{13}\sum_{i=1}^{50}C_{i,t+1}\left({\bar{a}}_{i,t}^{*}\right)}{\sum_{t=1}^{13}\sum_{i=1}^{50}C_{i,t+1}\left(0\right)}.$$

This population-level vaccine effectiveness is one minus the ratio of new cases under different vaccination speeds, similar to the definition of the usual vaccine effectiveness estimand [43]. This value can be interpreted as the proportion of new cases prevented by the vaccination speed ${\bar{a}}_{i,\;13}^{*}$ compared to the no-vaccination scenario.

### 2.4. Confounders

A sufficient set of confounders was adjusted to yield causal interpretation of our results. We adjusted the baseline cumulative number of cases and vaccination coverage. Based on the vaccine hesitancy framework and impact factors on vaccine uptake [27], we adjusted three types of confounders. The first type of confounder was related to people’s perception of risk and severity of COVID-19 pandemic; thus, we adjusted the lagged growth rate of new cases as an indicator of severity of the COVID-19 pandemic. It is a time-varying confounder. The second type of confounder was related to social influence from government and surrounding people. We used the overall government response index as an indicator of government behavior. We used vaccination coverage and vaccinated population (per 10,000) in the last week as indicators of surrounding people’s behavior. The confounders of the second type are also time-varying ones. For the third type of confounder, demographic and socio-economic factors at the state level were taken into consideration, including GDP, population, health resources (number of physicians), political position (red or blue states in the 2016 election), proportion of people aged 65 or above, racial composition, education level (proportion of people with advanced degrees), unemployment rate, and sex ratio.

### 2.5. Data Analysis

#### 2.5.1. Structural Nested Mean Model

Our analysis was mainly based on the structural nested mean model. The structural nested mean model parameterized the conditional average treatment effect:

(5) $$E\left[Y_{t+1}\left({\bar{a}}_{s},0\right)-Y_{t+1}\left({\bar{a}}_{s-1},0\right)|{\bar{L}}_{s}={\bar{l}}_{s},\;{\bar{Y}}_{s}={\bar{y}}_{s},\;\;{\bar{A}}_{s}={\bar{a}}_{s}\right],$$

where t=1,…,13, and s≤t. Following previous literature [15,16,17,18], we specified a linear model for the growth rate as

(6) $$E\left[Y_{t+1}\left({\bar{a}}_{s},0\right)-Y_{t+1}\left({\bar{a}}_{s-1},0\right)|{\bar{L}}_{s}={\bar{l}}_{s},\;{\bar{Y}}_{s}={\bar{y}}_{s},{\bar{A}}_{s}={\bar{a}}_{s}\right]=\psi a_{s}.$$

This SNMM specification was commonly used in past epidemiology literature [44,45]. Our SNMM could imply a marginal structural model (MSM) [46]:

(7) $$E\left[Y_{t+1}\left({\bar{a}}_{t}\right)\right]=E\left[Y_{t+1}\left(0\right)\right]+\psi \overset{t}{\underset{k=1}{\sum }}a_{k}.$$

Then, our causal parameter of interest could be represented as

(8) $$E\left[Y_{t+1}\left({\bar{a}}_{t}\right)-Y_{t+1}\left({\bar{a}}_{t}^{*}\right)\right]=\psi \overset{t}{\underset{k=1}{\sum }}\left(a_{k}-a_{k}^{*}\right).$$

In our dataset, $a_{k}$ represents the number of people out of every 10,000 people who were vaccinated for the first dose; then, $\sum_{k=1}^{t}a_{k}$ is the number of people who were vaccinated for the first dose since week 1. The parameter 100*ψ can be interpreted as the percentage of growth rate that decreases for every percent increase in the vaccinated population, starting from the first week.

To estimate ψ, we also specified a propensity score model (i.e., models for vaccinated population per 10,000 people) and an outcome model in each week to adjust for confounders. We utilized Poisson regression models for propensity scores and linear regression models for outcomes (Appendix B). ψ can be estimated using g-estimation (Appendix B) [36,37,38]. The standard error and 95% confidence interval were bootstrapped [47]. The estimation is doubly robust in the sense that the estimation for ψ is unbiased if either the propensity score models or the outcome models are correctly specified [38].

#### 2.5.2. Generalized Estimating Equation Models and Fixed Effects Models

We also fitted two generalized estimating equation (GEE) models [48,49] and two fixed effects models for comparison.

Two GEE models were fitted. The first GEE model regressed growth rate on the vaccinated population since week 1, adjusting baseline covariates. The second one regressed growth rate on the vaccinated population since week 1, adjusting baseline covariates, time-varying covariates, and time trend. We utilized the independent working correlation structure in our GEE analysis.

Two fixed effects models were also fitted. The first fixed effects model included state fixed effects, week fixed effects and vaccinated population since week 1. The second one included state fixed effects, week fixed effects, time-varying covariates, and vaccinated population since week 1. A detailed model specification of the GEE models and fixed effects models can be found in Appendix C.

#### 2.5.3. Scenarios Analysis

We also used the estimate from SNMM and g-estimation to predict the number of new cases each week in the United States under different vaccination speeds. For each state i, we utilized the following relationship to predict the counterfactual growth rate under vaccination speed ${\bar{a}}_{i,13}^{*}$:

(9) $$Y_{i,t+1}\left({\bar{a}}_{i,t}^{*}\right)=Y_{i,t+1}+\psi \overset{t}{\underset{k=1}{\sum }}\left(a_{i,k}^{*}-a_{i,k}^{✝}\right).$$

Here, $a_{i,k}^{*}$ is the k-th component of ${\bar{a}}_{13}^{*}$, and $a_{i,k}^{✝}$ refers to the observed vaccinated population in state i, week k. We predicted the counterfactual growth rate under the following hypothetical scenarios. In each scenario, we set a different vaccination speed.

- No-vaccination scenario: No people are vaccinated since week 1, which means ${\bar{a}}_{i,13}^{*}={\bar{0}}_{13}$.
- Twice speed scenario: The number of people vaccinated for the first time each week is twice the actual number in each state, which means ${\bar{a}}_{i,13}^{*}=\left(2a_{i,1}^{✝},2a_{i,2}^{✝},\ldots ,2a_{i,13}^{✝}\right)$.
- Half speed scenario: The number of people vaccinated for the first time each week is half of the actual number in each state, which means ${\bar{a}}_{i,\;13}^{*}=\left(\frac{a_{i,1}^{✝}}{2},\frac{a_{i,2}^{✝}}{2},\ldots ,\frac{a_{i,13}^{✝}}{2}\right)$.
- 1% constant speed scenario: 1% of the population receive their first dose in each week in each state, which means ${\bar{a}}_{i,\;13}^{*}=\left(100,\;100,\ldots ,\;100\right)$.
- 4% constant speed scenario: 4% of the population receive their first dose each week in each state, which means ${\bar{a}}_{i,\;13}^{*}=\left(400,\;400,\ldots ,\;400\right)$.
- Speed up scenario: For the first six weeks, 1% of the population receive their first dose in each week in each state, while for the remaining seven weeks, 4% of the population receive their first dose in each week in each state, which means ${\bar{a}}_{i,\;13}^{*}=\left(100,\;100,\ldots ,\;100,\;400,\ldots ,400\right)$.
- Speed down scenario: For the first seven weeks, 4% of the population receive their first dose in each week in each state, while for the remaining six weeks, 1% of the population receive their first dose in each week in each state, which means ${\bar{a}}_{i,\;13}^{*}=\left(400,\;400,\ldots ,\;400,\;100,\ldots ,100\right)$.

Under each counterfactual scenario, we utilized the relationship between growth rate and new cases to predict the number of new cases in each week, then calculated the population-level vaccine effectiveness.

#### 2.5.4. Additional Analysis and Extension

To demonstrate the validity of the method adopted, we conducted additional analyses and extended our statistical models to two new datasets. Firstly, we predicted the new cases in each scenario during the same study period with a modified Suspected–Infected–Recovered (SIR) model. Secondly, we used the SNMM, GEE models and fixed effects models described above to estimate the effect of vaccination on weekly growth rate based on the dataset from September 2021 to December 2021 in the United States when the delta variant dominated the epidemic. See Supplementary Material for more details.

## 3. Results

### 3.1. Baseline Characteristics

**Figure 1:**
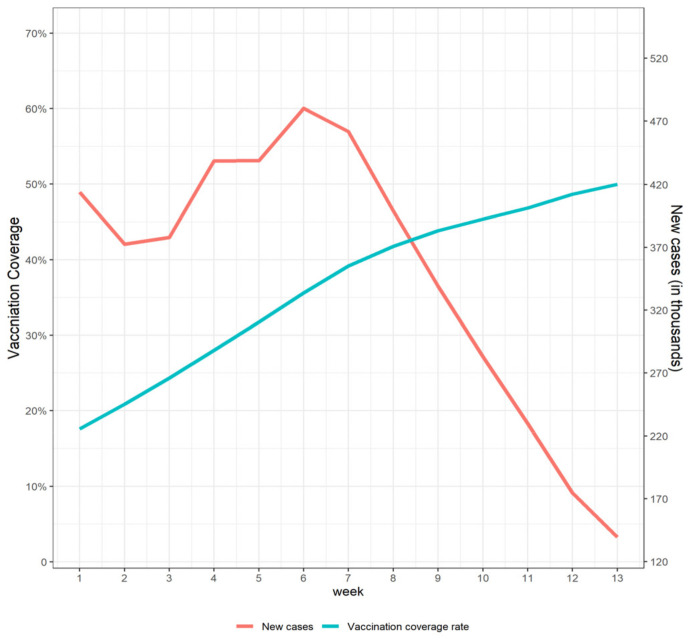
Vaccination coverage and new cases (in thousands) in the United States.

From 1 March to 30 May 2021, 4.55 million confirmed cases were reported in the US. The coverage rate of at least one dose among the general population reached 50.0% from 15.2% (Figure 1). The average weekly vaccination speed in the United States was 2.7%. Table 1 shows the descriptive statistics of the baseline covariates of 50 states.

### 3.2. Impact of COVD-I9 Vaccine Program on Weekly Growth Rate of COVID-19 New Cases

Table 2 shows the impact of the COVD-19 vaccination program on the weekly growth rate of new COVID-19 cases at the state level in the US. The result, based on the structural nested mean model (SNMM) with g-estimation, showed that for every 1% of the population becoming vaccinated, the growth rate of new COVID-19 infection cases reduced by 1.02% (95% CI: 1.69%, 0.26%). In contrast, the first GEE analysis with baseline covariates adjusted showed that every 1% increase in the COVID-19 vaccination coverage rate reduced the growth rate of new cases by 0.754% (95% CI: 0.974%, 0.533%), which was smaller than that of the g-estimation by approximately one standard error. The second GEE analysis, which adjusted the time trend and all covariates, estimated that every 1% increase in vaccination rate reduced the growth rate of new cases by 1.74% (95% CI: 2.42%, 1.05%), which was bigger than that of the g-estimation by approximately two standard errors. Results from the fixed-effects model showed similar patterns. The two-way fixed effects model showed that every 1% increase in the COVID-19 vaccination coverage rate reduced the growth rate of new cases by 1.52% (95% CI: 2.09%, 0.96%), which was bigger than that of the g-estimation by approximately 1.5 standard error. After adjusting for time-varying confounders, the two-way fixed effects model estimated that every 1% increase in the vaccination rate reduced the growth rate of new cases by 1.87% (95% CI: 2.43%, 1.30%) which was bigger than that of the g-estimation by approximately two standard errors.

### 3.3. Population-Level Effectiveness of COVID-19 Vaccination and Averted Disease Burden

**Figure 2:**
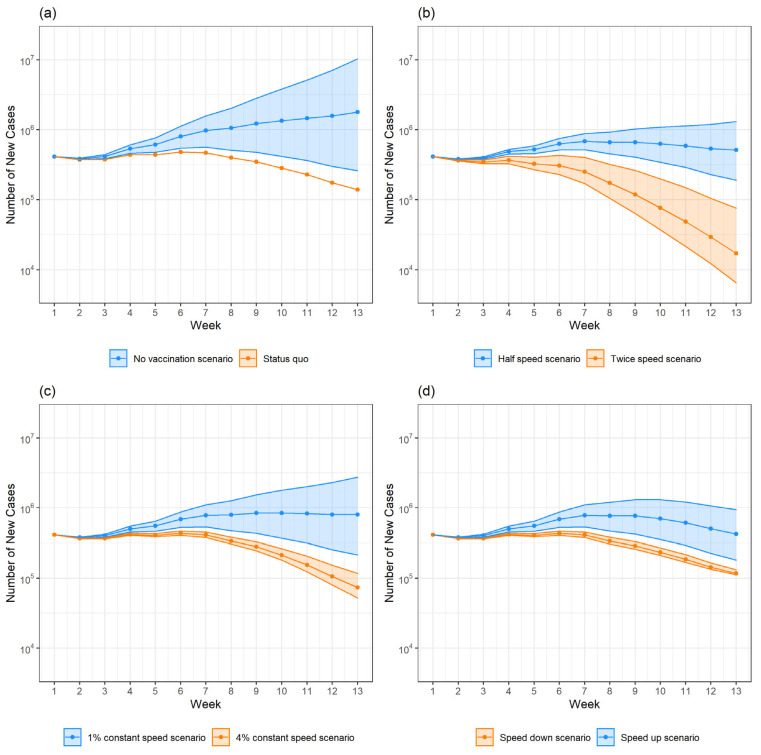
Predicted number of new cases in each week under different scenarios. (**a**) shows the comparison of predicted number of new cases under no vaccination scenario and status quo. (**b**) shows the comparison of predicted number of new cases under half speed scenario and twice speed scenario. (**c**) shows the comparison of predicted number of new cases under 1% constant speed scenario and 4% constant speed scenario. (**d**) shows the comparison of predicted number of new cases under speed up scenario and speed down scenario.

Table 3 shows the population-level effectiveness of COVID-19 vaccination and averted disease burden in total. Based on the causal analyses, if the COVID-19 vaccination had been suspended after the first week, the total infection cases during the study period would have reached 12.60 million (95% CI: 5.55, 36.51) in the US over the 13 weeks. By contrast, there were 4.55 million new cases under the status quo. At the present vaccination speed, the population-level effectiveness of COVID-19 vaccination was estimated as 63.9% (95% CI: 18.0%, 87.5%), and the COVID-19 vaccination program averted about 8 million cases. Figure 2a presents the comparison of cumulative cases over time between the status quo and the ‘no-vaccination’ scenario.

### 3.4. Scenarios Analysis

Table 3 and Figure 2b–d show the scenario analyses of the population-level effectiveness of COVID-19 vaccination and predicted disease burden of infection cases by different vaccination speeds.

In comparison to the status quo, doubling vaccination speed would have increased the vaccine effectiveness to 77.5% (95% CI: 29.2%, 93.6%), averting an additional 1.71 million cases. By contrast, if the vaccination program had been implemented at half the current speed, vaccination effectiveness would have declined to 43.7% (95% CI: 9.34%, 70.2%) with 2.55 million more infection cases. (Figure 2b).

As shown in Figure 2c, we predicted the results of the two scenarios where the vaccination programs were conducted at a constant speed of 1% or 4% of the general population per week. In the scenario with the speed of 1% of the population vaccinated per week, there would be 8.66 million cases (95% CI: 5.21, 16.06) with an estimated effectiveness of 31.3% (95% CI: 6.07%, 56.02%). In the scenario with the speed of 4% of the population vaccinated per week, there would be 3.99 million cases (95% CI: 3.71, 4.40) with an effectiveness of 68.4% (95% CI: 20.7%, 89.8%).

In the last set of comparisons (Figure 2d), we compared the results of ‘speed-up‘ and ’speed-down‘ scenarios, keeping the total amount of vaccination during the 13 weeks basically the same as that of the status quo (34.8%). In the ’speed-down‘ scenario, where the vaccination speed was 4% of the population per week for the first seven weeks and 1% for the last six weeks, vaccination speed changed from fast to slow. There would be 4.13 million cases (95% CI: 3.91, 4.44) with the vaccine effectiveness being approximately 67.3% (95% CI: 19.9%, 89.3%). However, if the vaccination speed had been 1% of the population per week for the first six weeks and 4% for the last seven weeks, vaccination speed changed from slow to fast. The total cases would have reached 7.52 million cases (95% CI: 5.10, 11.47), and the estimated vaccine effectiveness would have declined to 40.3% (95% CI: 8.10%, 68.6%).

## 4. Discussion

The structural nested mean model examined the causal relationship between COVID-19 vaccination coverage and the growth rate of new infection cases at the population level. From 1 March to 30 May 2021, for every 1% of the population becoming vaccinated, the growth rate of new COVID-19 infection cases was reduced by 1.02% (95% CI: 0.26%, 1.69%) in the US. In comparison to a no-vaccination scenario, the current COVID-19 vaccination program averted 8 million infection cases, with an estimated vaccine effectiveness of 63.9% (95% CI: 18.0%, 87.5%). Our results show that intensive vaccination rollout, especially at the early stages of the vaccination campaign, is crucial for reducing infections.

The real-world vaccine effectiveness at the individual level has been studied, which typically focuses on the vaccine’s direct effectiveness [14]. For example, the vaccine effectiveness of BNT162b2 or mRNA-1273 COVID-19 vaccines in preventing SARS-CoV-2 infection was 90% for full immunization and 80% for partial immunization in the US [8]. The case-control study in England estimated the effectiveness of BNT162b2 to be 70% in 10 to 13 days after the first dose and 89% in 14 days after the second dose [11]. The cohort study in Sweden showed that the estimated effectiveness of BNT162b2 in preventing infection >7 days after the second dose was 86%, but only 42% for >14 days after a single dose [7]. Our estimate at the population level was smaller than that at the individual level for the following reasons. First, it should be noted that vaccine effectiveness at the population level demonstrated the effect of the general vaccine program, which is conceptually different from that at the individual level [14]. The population-level effectiveness was estimated by comparing the effect of vaccination programs, at different vaccination speeds and in different states, on the infection. In comparison, the individual-level effectiveness was estimated by comparing the risks of vaccinated and non-vaccinated individuals. Secondly, the population-level effectiveness would be influenced by the change in population vaccination coverage at a given period of time. The increment of vaccination coverage was approximately 35% in our study period. The population-level effectiveness might be larger if a longer period were examined.

So far, a few studies have examined the population-level effectiveness of COVID-19 vaccines using an agent-based model. Moghadas et al. [20] used an agent-based model and found that compared to the no-vaccination scenario, a vaccination campaign achieving 40% coverage of the entire population within 284 days would reduce cases by 50% [20]. Their increment of vaccination coverage was close to that of our study period (approximately 35%) and their result was also close to our estimated 63.9% effectiveness. Their agent-based model depicted the whole disease dynamics, which required strong modeling assumptions about how individuals transit between different states. The simulations relied heavily on pre-estimated model parameters. Our causal approach, on the other hand, focused more on the relationship between independent and outcome variables and retrospectively utilized real data during the study period, including vaccination coverage data, epidemic data and other covariates.

When handling longitudinal data to estimate the population-level effectiveness of the COVID-19 vaccines, it is essential to address the issue of potential time-varying confounders, which may be impacted by the treatment (vaccinated population) at the prior period. The use of SNMM helped to address the issue correctly. In comparison, the GEE model adjusting baseline covariates and the two-way fixed effects models could produce biased estimates because they did not adjust the time-varying confounders. Therefore, we found that their results differed from the estimate of SNMM by at least one standard error. The GEE model adjusting baseline and time-varying confounders and the two-way fixed effects models adjusting time-varying confounders could produce biased estimates because they adjusted time-varying confounders by directly conditioning on those confounders, which would produce collider bias [30,31,33,50]. This bias may explain why their results differed from the estimate of SNMM by two standard errors. Similarly, SIR models did not adjust any confounders, which may account for the difference between the prediction by SNMM and the prediction by the SIR model. Moreover, based on the potential outcome framework, our analyses allowed us to clearly define causal estimands without ambiguity and thus enable a causal interpretation. The causal framework is advocated in evaluating vaccine performance, including efficacy and effectiveness [51]. Unlike other causal methods for assessing vaccine effectiveness, such as the synthetic control method and the fixed effects model, our model could study the causal effect of the continuous treatments under a quickly evolving and dynamic context, especially in the current COVID-19 pandemic.

One key assumption for causal interpretation is sequential ignorability [36,46]. To make this assumption hold, all confounders between treatments and outcomes in each stage must be observable and properly adjusted. The literature on vaccine hesitancy helped in determining the set of confounders that needed to be adjusted [27]. We included a sufficiently large set of variables based on the vaccine hesitancy framework to ensure that confounders were adequately adjusted in this context. Another key assumption for causal interpretation is consistency [36,46], which implicated no hidden versions of treatments [52]. A possible violation of this assumption may be due to the different types of COVID-19 vaccines used for the vaccination campaign, because their efficacy varies [4,5,6], but this problem is negligible because the Johnson and Johnson vaccine was only used by a small proportion of the population (approximately 6%) [3]. The two widely-used types of vaccines (Pfizer-BioNTech and Moderna) showed similar efficacy in randomized clinical trials (94.1% vs. 95%) [4,5].

This study quantified the impact of vaccination speed on vaccine effectiveness and disease burden averted. Compared with the status quo, an additional 1.71 million cases could be averted if the vaccination program were launched at twice the actual vaccination speed; an additional 0.56 million cases could be averted if the vaccination program were launched at a constant speed of 4% of the population for each week. Simulations from mathematical models also yielded similar results. A study found that cumulative mortality would be 442, 241, and 50 (per 100,000) for daily vaccination rates of 0.1%, 0.3%, and 1% respectively [24]. Another study found that accelerating vaccine delivery would substantially reduce severe health outcomes [53].

Many countries around the world have made plans to achieve a specific vaccine coverage rate at a specific time point. For example, the U.S. government planned to achieve a goal of 70 percent vaccine coverage by 4 July 2021 [54]. The South Korean government planned to achieve a goal of 70 percent vaccine coverage by the end of the third quarter of September [55]. However, there have not been suggestions regarding targeting vaccine coverage rates at each stage over the course of the vaccination campaign. The simulated scenarios, especially the ‘speed up’ and ‘speed down’ scenarios, help to demonstrate the importance of accelerating the vaccination rollout in the early stages. First, the comparison between the ‘speed up’ scenario and ‘speed down’ scenario shows that even if the total increment of the vaccine coverage is the same over a period of the campaign, the rate of vaccination at each stage (e.g., week) of the period can still have a significant impact on the population-wide effectiveness of the vaccine. If the vaccination coverage increment over the 13 weeks is the same (approximately 35%) as the status quo, the ‘speed up’ scenario, which has a lower vaccination speed at the early stage, would cause 3.13 million additional cases compared to the ‘speed down’ scenario. Second, we found that the ‘speed up’ scenario was significantly worse than the ‘status quo’, as the 95% confidence interval of cases under ‘speed up’ (5.10 million to 11.47 million) does not cover the cases under the status quo (4.55 million). Third, we found that the ‘speed down’ scenario was significantly better than the ‘status quo’ because the 95% confidence interval of cases under ‘speed down’ (3.91 million to 4.44 million) did not cover the cases under the status quo (4.55 million) either. In addition, from a scale perspective, the ‘speed down’ scenario could reduce cases by an additional 0.42 million, which equals 10% of the status quo cases during the period. In fact, the status quo was similar to the ‘speed down’ where vaccination speed declined gradually over time, because the average speed of vaccination under the status quo was higher for the first seven weeks than for the last six weeks, which means reality also followed the pattern that the vaccination speed was faster in the early stage. This fact explains why the ‘speed down’ curve in Figure 2 is closer to status quo.

Our results have several important policy implications. First, increasing vaccination coverage is critical for reducing infections. By the end of May, the proportion of people vaccinated reached 50% in the United States, and the incidence of COVID-19 continued to decline. In many other countries and regions, however, the vaccination coverage rate is still low. For example, by the end of May, only 2 doses of COVID-19 vaccines were administered per 100 people in Africa (in comparison, the United States had administered 93 doses per 100 people), and Africa was facing a fast-surging third wave of the COVID-19 pandemic at the same time [56]. Second, accelerating the vaccination speed at the early stage is crucial for reducing infections. Therefore, interventions should be conducted from the beginning of vaccination campaigns to increase the vaccination speed, including approaches such as scaling up manufacturing, promoting effective deployment and avoiding unnecessary waste, and health education to eliminate vaccine hesitancy. These measures are also critical for the current period, given the spread of new COVID-19 variants and the need for booster shots in the US and other countries.

Our paper has several limitations. First, our structural nested mean model assumes a constant effect of vaccinated population on subsequent growth rate during the study period for the convenience of explaining the parameter. Although this model specification has also been widely adopted in other research based on longitudinal causal inference methods [44,46], non-constant treatment effect may occur with changes in vaccination coverage. Second, due to different epidemic patterns of the delta variant, the present results only report the vaccine effectiveness against the original variants based on data from March to May. Future studies could extend the structural nested mean models (SNMMs) to examine the effect of COVID-19 vaccination programs against other variants.

## 5. Conclusions

The COVID-19 vaccination program demonstrated a high level of effectiveness at the population level and significantly reduced the disease burden in the United States. We quantified the impact of vaccination speed on the effectiveness of the COVID-19 vaccination program and the disease burden averted. Intensive vaccination rollout, especially at the early stages of the vaccination campaign, is crucial for reducing COVID-19 infections.

## Figures and Tables

**Table 1:** Descriptive statistics of baseline covariates.

Variables	Mean	SD
Number of physicians (per capita)	475.2	192.6
GDP (millions of chained 2012 dollars)	375,880	490,590
Population	6,551,748	7,415,328
Race composition (proportion of black people)	0.132	0.109
Proportion of old people (aged 65 or above)	0.164	0.020
Red or Blue state in 2016 election (Red = 1)	0.6	0.495
Unemployment Rate (in March, 2021)	5.56	1.72
Proportion of people with advanced degrees	0.126	0.042
Sex ratio	0.977	0.033
Cumulative cases at baseline	569,301	664,218
Vaccination coverage at baseline (per 10,000 people)	1572	238

**Table 2:** Impact of the COVD-19 vaccine program on the weekly growth rate of new COVID-19 cases.

	Decline of Growth Rate		
	Estimate	SE	95% CI
Main analysis			
SNMM with g-estimation	1.02%	0.0037	(1.69%, 0.26%)
GEE analysis			
GEE (adjust baseline covariates)	0.754%	0.00076	(0.974%, 0.533%)
GEE (adjust baseline and time-varying covariates)	1.74%	0.0035	(2.42%, 1.05%)
Fixed effects model			
Two-way fixed effects model	1.52%	0.0029	(2.09%, 0.96%)
Two-way fixed effects model (adjust time-varying covariates)	1.87%	0.0029	(2.43%, 1.30%)

**Table 3:** Results of the base case and scenario analyses.

Scenarios	Cumulated New Cases (Million)		Vaccination Effectiveness	
	Estimate (95% CI)	Difference (%) ^a^	Estimate (95% CI)	Difference (%) ^b^
Base case				
Status quo	4.55 ^c^	/	63.9% (18.0%, 87.5%)	/
Scenario analysis				
No-Vaccination	12.60 (5.55, 36.51)	8.05 (177%)	0%	−63.9%
vaccination speed: two times the status-quo speed	2.84 (2.34, 3.93)	−1.71 (−37.6%)	77.5% (29.2%, 93.6%)	13.6%
vaccination speed: half of the status-quo speed	7.10 (5.03, 10.88)	2.55 (56.0%)	43.7% (9.34%, 70.2%)	−20.2%
vaccination speed: 4% population per week	3.99 (3.71, 4.40)	−0.56 (−12.3%)	68.4% (20.7%, 89.8%)	4.5%
vaccination speed: 1% population per week	8.66 (5.21, 16.06)	4.11 (90.3%)	31.3% (6.07%, 56.02%)	−32.6%
Speed-down: 4% for first 7 weeks and 1% for last 6 weeks	4.13 (3.91, 4.44)	−0.42 (−9.2%)	67.3% (19.9%, 89.3%)	3.4%
Speed-up: 1% for first 6 weeks and 4% for last 7 weeks	7.52 (5.10, 11.47)	2.97 (65.3%)	40.3% (8.10%, 68.6%)	−23.6%

^a.^ The difference is the estimate of cases under each scenario minus the cases under the status quo. The percentage in the bracket is the ratio of difference over cases under the status quo. ^b.^ The difference refers to estimates of vaccine effectiveness in each scenario minus the effectiveness under the status quo. ^c.^ The observed cases under the status quo.

## Data Availability

Publicly available datasets were analyzed in this study. This data can be found here: https://github.com/wangrui24/COVID_Vaccine_paper (accessed on 5 April 2022).

## Supplementary Materials

The following are available online at https://www.mdpi.com/article/10.3390/vaccines10050726/s1, Figure S1: Directed Acyclic Graph (DAG), Figure S2: Simulating the SIR model with σ = 0, Figure S3: Simulating the SIR model with σ = 0.05, Figure S4: Simulating the SIR model with σ = 0.15, Figure S5: Simulating the SIR model with σ = 0.25, Figure S6: Simulating the SIR model with σ = 0.35, Figure S7: Simulating the SIR model with σ = 0.45, Figure S8: The vaccination coverage and new cases (in thousands) in the United States (from 5 September 2021 to 4 December 2021), Table S1: Impact of COVD-I9 vaccine program on weekly growth rate of COVID-19 new cases, Figure S9: Predicted Number of new cases in each week under different scenarios (September 2021–December 2021), Table S2: Results of the base case and scenarios analyses (September 2021–December 2021).

## Appendix A

**Table A1:** Data sources.

Variables	Value Ranges	Source, Period of Data Collection
Number of physicians (per capita)	Not Applicable	https://www.fsmb.org/siteassets/advocacy/publications/2018census.pdf 2018 Accessed on 6 June 2021
GDP (millions of chained 2012 dollars)	Not Applicable	https://apps.bea.gov/regional/histdata/releases/0720gdpstate/index.cfm 2019 Accessed on 6 June 2021
Population	Not Applicable	https://apps.bea.gov/regional/histdata/releases/0320sqpi/index.cfm Fourth Quarter 2019 Accessed on 6 June 2021
Racial composition (proportion of black people)	0–1	http://www.census.gov/library/visualizations/interactive/race-and-ethnicity-in-the-united-state-2010-and-2020-census.html 2019 Accessed on 6 June 2021
Proportion of old people (aged 65 or above)	0–1	https://acl.gov/sites/default/files/Aging%20and%20Disability%20in%20America/2019ProfileOlderAmericans508.pdf 2019 Accessed on 6 June 2021
Red or Blue state in 2016 election (Red = 1)	0 and 1	https://www.fec.gov/resources/cms-content/documents/federalelections2016.pdf 2016 Accessed on 6 June 2021
Unemployment Rate	Not Applicable	https://www.ncsl.org/research/labor-and-employment/state-unemployment-update.aspx/ March, 2021 Accessed on 10 Novermber 2021
Proportion of people with advanced degrees	0–1	https://acl.gov/sites/default/files/Aging%20and%20Disability%20in%20America/2019ProfileOlderAmericans508.pdf 2019 Accessed on 10 Novermber 2021
Sex ratio	Not Applicable	https://worldpopulationreview.com/state-rankings/male-to-female-ratio-by-state 2021 Accessed on 10 Novermber 2021

## Appendix B. Details about the Estimation of SNMM

In this paper, we adopted the estimation procedure proposed by Vansteelandt [47]. The estimation procedure is described in the following steps:

Step 1: We fit a pooled Poisson regression model for vaccination population (per 10,000 people) in each week:

$$\begin{array}{cc} E\left[A_{i,t}|{\bar{A}}_{i,t-1},{\bar{L}}_{i,t},{\bar{Y}}_{i,t}\right] & \\ & =exp(\alpha_{0}+\alpha_{1}t+\alpha_{1}t^{2}+\alpha_{3}{physicans}_{i}+\alpha_{4}{election}_{i}+\alpha_{5}{GDP}_{i}+\alpha_{6}{race}_{i}\\ & +\alpha_{7}{old}_{i}+\alpha_{8}{populatiin}_{i}+\alpha_{9}{cumcasei0}_{i}+\alpha_{10}{basvac}_{i}+\alpha_{11}{education}_{i}\\ & +\alpha_{12}{sexratio}_{i}+\alpha_{13}{unemployment}_{i}+\alpha_{14}Y_{it}+\alpha_{15}\overset{t-1}{\underset{k=1}{\sum }}A_{ik}+\alpha_{16}A_{it-1}\\ & +\alpha_{17}{goverment}_{it}) \end{array}$$

$A_{i,t}$ is the independent variable, the vaccinated population (per 10,000 people) in state i at week t.

$Y_{it}$ is the outcome variable, the growth rate of new cases in state i at week t. $L_{it}$ denotes other confounders in state i at week t.

$physicans_{i}$ is number of physicians (per capita) in state i.

$election_{i}=1$ denotes state i is red state in 2016 president election.

$GDP_{i}$ is the GDP (millions of chained 2012 dollars) in state i.

$race_{i}$ is the racial composition (proportion of black people) in state i.

$old_{i}$ is the proportion of old people (aged 65 or above) in state i.

$pop_{i}$ is the population in state i.

$cumcase0_{i}$ is the cumulated confirmed cases at baseline in state i.

$basvac_{i}$ is the vaccinated population at baseline in state i.

$education_{i}$ is the education level (Proportion of people with advanced degrees) in state i.

$sexratio_{i}$ is the sex ratio in state i.

$unemployment_{i}$ is the unemployment rate in state i.

$government_{it}$ is the government response index at week t in state i.

Step 2: Predict the fitted value of $A_{it}$, denote it as $p_{it}$.

Step 3: Fit the following linear regression:

$$\begin{array}{cc} E\left[Y_{i,t+1}|{\bar{A}}_{i,t},{\bar{L}}_{i,t},{\bar{Y}}_{i,t}\right] & \\ & =\beta_{0}+\beta_{1}t+\beta_{1}t_{2}+\beta_{3}{physicans}_{i}+\beta_{4}election_{i}+\beta_{5}GDP_{i}\\ & +\beta_{6}race_{i}+\beta_{7}old_{i}+\beta_{8}population_{i}+\beta_{9}cumcase0_{i}+\beta_{10}basvac_{i}\\ & +\beta_{11}education_{i}+\beta_{12}sexratio_{i}+\beta_{13}unemployment_{i}+\beta_{14}Y_{is}\\ & +\beta_{15}\overset{t-1}{\underset{k=1}{\sum }}A_{k}+\beta_{16}A_{i,t-1}+\beta_{17}government_{it}+\beta_{18}p_{it}+\psi A_{it} \end{array}$$

We can obtain a preliminary estimate of ψ, denoted ψ ^0, through this regression.

Step 4: Predict the potential outcome:

Let

$$H_{i,t+1,s+1}=Y_{i,t+1}-{\hat{\psi }}^{0}\overset{t}{\underset{k=s+1}{\sum }}A_{ik}$$

where s ranges from 1 to t−1, and $H_{i,t+1,s+1}$ can be viewed as a mimic of $Y_{i,t+1}\left(A_{1},\ldots ,A_{s},0,\ldots ,0\right)$.

Step 5: Fit the following linear regression:

We can obtain an updated estimate of ψ, denoted ${\hat{\psi }}^{1},$ through this regression.

$$\begin{array}{cc} E\left[H_{i,t+1,s+1}|{\bar{A}}_{i,s},{\bar{L}}_{i,s},{\bar{Y}}_{i,s}\right]\\ & =\beta_{0}+\beta_{1}s+\beta_{2}s^{2}+\beta_{3}physicans_{i}+\beta_{4}election_{i}+\beta_{5}GDP_{i}\\ & +\beta_{6}race_{i}+\beta_{7}old_{i}+\beta_{8}population_{i}+\beta_{9}cumcase0_{i}+\beta_{10}basvac_{i}\\ & +\beta_{11}education_{i}+\beta_{12}sexratio_{i}+\beta_{13}unemployment_{i}+\beta_{14}Y_{is}\\ & +\beta_{15}\overset{s-1}{\underset{k=1}{\sum }}A_{k}+\beta_{16}A_{i,s-1}+\beta_{17}government_{is}+\beta_{18}p_{is}+\psi A_{is} \end{array}$$

Step 6: Using the updated estimate of ψ to repeat Step 4 to Step 5 several times [47], we can obtain a final estimate of ψ.

## Appendix C. Specification of GEE Models and Fixed Effects Models

GEE (adjust baseline covariates):

$$\begin{array}{cc} E\left[Y_{i,t+1}|{\bar{A}}_{i,t},{\bar{L}}_{i,t},{\bar{Y}}_{i,t}\right] & \\ & =\gamma_{0}+\gamma_{3}physicans_{i}+\gamma_{4}election_{i}+\gamma_{5}GDP_{i}+\gamma_{6}race_{i}\\ & +\gamma_{7}old_{i}+\gamma_{8}population_{i}+\gamma_{9}cumcase0_{i}+\gamma_{10}basvac_{i}\\ & +\gamma_{11}education_{i}+\gamma_{12}sexratio_{i}+\gamma_{13}unemployment_{i}\\ & +\delta \overset{t}{\underset{k=1}{\sum }}A_{ik} \end{array}$$

GEE (adjust baseline covariates and time-varying covariates):

$$\begin{array}{cc} E\left[Y_{i,t+1}|{\bar{A}}_{i,t},{\bar{L}}_{i,t},{\bar{Y}}_{i,t}\right] & \\ & =\gamma_{0}+\gamma_{1}t+\gamma_{2}t^{2}+\gamma_{3}physicans_{i}+\gamma_{4}election_{i}+\gamma_{5}GDP_{i}\\ & +\gamma_{6}race_{i}+\gamma_{7}old_{i}+\gamma_{8}population_{i}+\gamma_{9}cumcase0_{i}\\ & +\gamma_{10}basvac_{i}+\gamma_{11}education_{i}+\gamma_{12}sexratio_{i}\\ & +\gamma_{13}unemployment_{i}+\gamma_{14}Y_{it}+\gamma_{17}government_{it}\\ & +\delta \overset{t}{\underset{k=1}{\sum }}A_{ik} \end{array}$$

Two-way fixed effects model:

$$Y_{it}=\mu_{i}+\theta_{t}+\delta \overset{t}{\underset{k=1}{\sum }}A_{ik}+\varepsilon_{it}$$

where $\mu_{i}$ is the state fixed effect, $\theta_{t}$ is the week fixed effect, $\varepsilon_{it}$ is the error term.

Two-way fixed effects model (adjust time-varying covariates):

$$Y_{it}=\mu_{i}+\theta_{t}+\gamma_{12}Y_{it}+\gamma_{13}government_{it}+\delta \overset{t}{\underset{k=1}{\sum }}A_{ik}+\varepsilon_{it}$$

In Table 2, we reported the estimate of δ.
