# Supplementary material for: Population-Level Effectiveness of COVID-19 Vaccination Program in the United States: Causal Analysis Based on Structural Nested Mean Model
Source: Vaccines (Basel). 2022 May 5;10(5):726. doi: 10.3390/vaccines10050726 (PMC9144931; doi:10.3390/vaccines10050726)
Supplement: Supplementary file 1 [file vaccines-10-00726-s001.zip › vaccines-1525778-supplementary.pdf]

Supplementary Material S1.: A Simulation Study on COVID-19 Vaccination Based on an SIR Model

In this section, we modified an SIR model and proposed a sensitivity analysis method to adjust contact rate under different scenarios to predict the case under each scenario and make comparisons with the results of SNMM. Supplementary material 1 is organized as follows:

- In the first section, we explain the longitudinal structural and time-varying confounding problem in detail.
- In the second section, we explain how the time-varying confounders could have an impact on the prediction of SIR under different scenarios.
- In the third section, we develop a sensitivity analysis method for the SIR model to adjust for the time-varying confounding problem. Our results show that under certain settings, the prediction of the SIR model is similar to the prediction of SNMM.

1. The Longitudinal Structure and Time-Varying Confounding Problem

To understand how time-varying confounders could have an impact on the SIR model, in this section we revisit the longitudinal structure and time-varying confounding problem. The time-varying confounding problem could be illustrated using a directed acyclic graph (DAG, Figure S1) [1]:

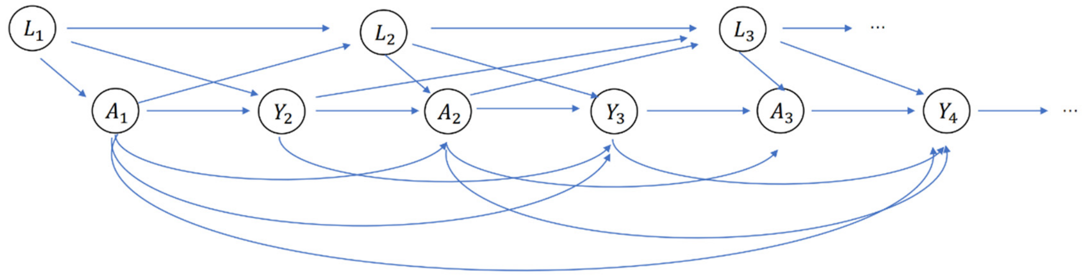

Figure S1. Directed Acyclic Graph (DAG): This DAG shows the longitudinal structure of observed data. A_t represents the treatment (i.e. the number of people (per 10,000) who got their first dose) at week t. Y_t represents the growth rate of new COVID-19 cases for week t. L_t represents the confounders we observed at time t, including the baseline confounders and time-varying confounders.

The notations are consistent with those in the manuscript. The subscript t refers to the time (week). Treatment (denoted as A_t) represents the number of people (per 10,000) who got their first dose at week t. The outcome variable Y_t represents the growth rate of new COVID-19 cases for each week. L_t represents the confounders we observed at time t, including the baseline confounders and time-varying confounders. As shown in the DAG, the vaccination and confounders (e.g., the government response index, GDP) could have impacts on the outcome variables (growth rate of new cases) of the subsequent periods.

To address the time-varying confounding problem, we adjust three kinds of time-varying confounders in our SNMM based on the literature on vaccine hesitancy and impact factors on vaccine uptake [2]: the government response index (which represents the behavior of government), the vaccination coverage (which represents the behavior of residents) and the growth rate of new cases from the previous period (which represents the severity of pandemic). Those time-varying confounders are also called treatment-induced confounders since they are descendants of treatment variables, so they can also be written as potential outcomes. For example, L_3 is descendant of (A_1, A_2), so if we set $(A_1, A_2) = (a_1, a_2)$, the potential outcome we would have observed should be $L_3(a_1, a_2)$. The SNMM with g-estimation could properly adjust the time-varying confounding problem, which is

an advantage over traditional regression-based models (i.e. GEE and fixed effects models) as we mentioned in the manuscript.

2. The Impact of Time-Varying Confounders on the SIR Model

In this section, we describe the impact of the time-varying confounding problem on the SIR model.

To the best of our knowledge, the existing SIR models and their variants can't be incorporated into the causal framework since they cannot handle confounders (including baseline confounders and time-varying confounders). As a result, the simulation of SIR models only gives predictions based on the association in observed data and lacks causal interpretation due to the failure of adjusting confounders. The SIR model estimated using real data can only represent the data generating mechanism under the status quo rather than the data generating mechanism under counterfactual scenarios. Hence, we would produce biased estimates if we use the SIR model estimated using real data to predict the cases in counterfactual scenarios. Here are the reasons:

- As we stated in the first section, the time-varying confounders can be written as the potential outcome of past treatments $\bar{a}_t$ (i.e. the vaccinated population at each week, or we called the vaccination speed in the manuscript): $L_{t+1}(\bar{a}_t)$.
- The key parameter β_{t+1} of the SIR model, the contact rate, is a function of those time-varying confounders (which represent the behavior of government and residents) [3]. Therefore, β_{t+1} can also be written as the potential outcome of past treatments: $\beta_{t+1}(\bar{a}_t)$.
- The purpose of the scenario analyses was to estimate how the number of cases would change under various counterfactual vaccination scenarios. Therefore, in each scenario, we need to set different $\bar{a}_t$ as hypothetic interventions. Since $\beta_{t+1}(\bar{a}_t)$ can also be written as the potential outcome of past treatments, it will be different under different scenarios. For example, in the no vaccination scenario, the $\bar{a}_{13}$ is set as $(0,0,\ldots,0)$, which is different from the vaccination speed under the status quo. Therefore, the parameter β_{t+1} under no vaccination scenario (we can denote as $\beta_{t+1}(\bar{0}_t)$) could be different from the parameter β_{t+1} under status quo (we can denote it as $\beta_{t+1}(\bar{a}_t^{\dagger})$, here $\bar{a}_t^{\dagger}$ represents the vaccination speed under status quo, which is observable).
- To sum up, under each counterfactual scenario, we could have different β_{t+1} for the SIR models. And when the vaccination speed is set as $\bar{a}_t$, theoretically, we should simulate the cases using the SIR model with parameters $\beta_{t+1}(\bar{a}_t)$. However, since the functional form of $\beta_{t+1}(\bar{a}_t)$ is unknown, we can only use the β_{t+1} estimated from the real data to simulate the cases under counterfactual scenarios in previous versions of our simulations. The difference between $\beta_{t+1}(\bar{a}_t^{\dagger})$ and $\beta_{t+1}(\bar{a}_t)$ could explain why the curves in Figure S2 are quite different than the corresponding ones in Figure 2: The difference in estimated cases between SNMM and SIR may be due to the failure of adjusting time-varying confounders in the SIR model, which will lead to a wrong β_{t+1} under counterfactual scenarios.

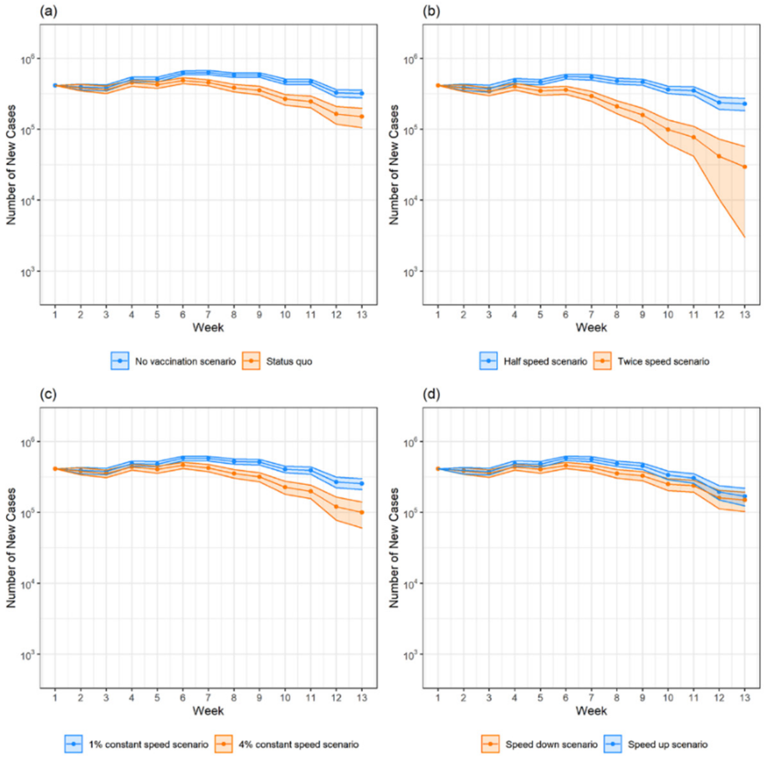

Panel A: Predicted number of new cases in each week under different scenarios using SIR model.

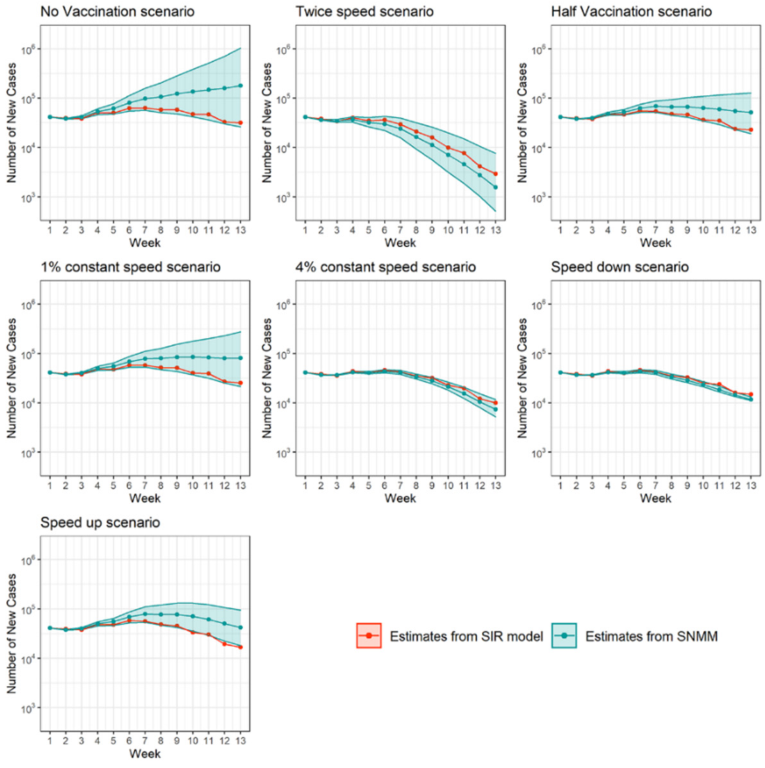

Panel B: Comparisons of predicted new cases between SIR model and SNMM.

Figure S2. Simulating the SIR model with $\sigma = 0$. Panel A shows the predicted number of new cases in each week under different scenarios using SIR model: (**a**) shows the comparison of predicted number of new cases under no vaccination scenario and status quo. (**b**) shows the comparison of predicted number of new cases under half speed scenario and twice speed scenario. (**c**) shows the comparison of predicted number of new cases under 1% constant speed scenario and

4% constant speed scenario. (**d**) shows the comparison of predicted number of new cases under speed up scenario and speed down scenario. Panel B shows the comparison between SIR model and SNMM. The green area is the 95% CI of SNMM.

3. A Modified SIR Model and Sensitivity Analysis

To alleviate the bias of SIR model caused by the time-varying confounders, we modified an SIR model under the status quo and proposed a sensitivity analysis method to adjust for $\beta_{t+1}(\bar{a}_t)$ under different scenarios in this section. Our results show that, after adjusting for the $\beta_{t+1}(\bar{a}_t)$ under different counterfactual scenarios, the predictions of the SIR model under different scenarios can be similar to the predictions of SNMM.

3.1. The SIR Model under Status Quo

The modified SIR model is one of the state-space compartment models consisting of S (susceptible) for those who are susceptible to SARS-CoV-2, I (infected) for those who are infected and contagious, and R (removed) for those who have recovered or died from the disease or those who have developed immunity with SARS-CoV-2 (e.g., vaccinated people). For convenience, we assume all the infections will be confirmed by official institutes. The dynamic equation can be expressed by a group of ordinary differential equations as Equation (S.1).

$$\begin{aligned} \frac{\mathrm{d}S}{\mathrm{d}t} &= -\beta(t)\ \frac{S(t)}{N}I(t) - V(t), \\ \frac{\mathrm{d}I}{\mathrm{d}t} &= \ \beta(t)\frac{S(t)}{N}I(t) - \gamma(t)I(t), \\ \frac{\mathrm{d}R}{\mathrm{d}t} &= \gamma(t)I(t) + V(t), \end{aligned} \qquad \text{(S.1)}$$

where $\beta(t)$ is the product of the effective contact number per head and the transmission probability of each effective contact, representing the transmission rate of the SARS-CoV-2, $\gamma(t)$ is the reciprocal of the average time from infection to recovery or death, N is the overall population of the US, and $V(t)$ is the added size in population immunity for day t, where we approximate it with the number of daily vaccinated people for the first dose during the previous week [4]. The approximation of $V(t)$ is supported by evidence that the vaccinated people will get immuned after 7 days [5]. For simplification, we let $\beta(t)$ be constant every two weeks, denoted as $\beta^{(1)}$ to $\beta^{(6)}$, while $\gamma(t)$ be constant $\gamma(t)$ from week 2 to week 13.

For the estimation of unknown parameters, we utilize the observed number of weekly confirmed cases and estimate the parameters β and γ with maximum likelihood estimation (MLE). For the t-th week, the conditional mean of the confirmed case should be $\int_t^{t+1} \beta_k \frac{I_k S_k}{N}\, dk$, denoted as C_t (Here we use X(t) denote daily level value of variable X, X_t denote the weekly level value of variable X). The observed number of confirmed cases for the t-th week ${C^{ob}}_t$ approximately follows a Poisson distribution with mean as C_t. The likelihood can be denoted by $\prod_{t=1}^{T} Po({C^{ob}}_t | C_t)$ and we estimate the parameters by maximizing the logarithm of likelihood. We use the weekly confirmed cases from Mar 8, 2021, to May 30, 2021 to fit the model [6]. The initial value for the dynamic equations is set to be the epidemic status on Mar 7, 2021. The initial value for $I(t)$, denoted as $I(0)$, is the observed cases currently infected on Mar 7, 2021 retrieved from Worldometers [7]. The initial value for $R(t)$, denoted as $R(0)$, is imputed by the cumulative recovery and deaths plus the overall vaccinations for at least one dose up to Mar 7, 2021. The initial value for $S(t)$ is computed as $N - I(0) - R(0)$. In our study, the initial value $(S(0), I(0),\ R(0))$ is set as $(249{,}999{,}014,\ \ 7{,}505{,}438,\ \ 70{,}082{,}950)$. We optimize the log-likelihood with Newton-Raphson method.

We then simulate the epidemic curve under different scenarios of vaccination based on estimated parameters with random perturbation. We induce the stochasticity to our modified SIR model by using a multinomial distribution as perturbation, i.e., consider the dynamic number of day $t+1$ computed by Equation (S.1) as $(\tilde{S}(t+1), \tilde{I}(t+1), \tilde{R}(t+$

$1)\big)$, and we randomly sample the epidemic for day $t+1$ from $(S(t+1), I(t+1), R(t+1)) \sim \text{Mult}\big(N, \big(\tilde{S}(t+1), \tilde{I}(t+1), \tilde{R}(t+1)\big)/N\big)$ [8]. We repeat the simulation 1,000 times for each vaccination scenario and obtain the sample mean as the estimation for the time-varying epidemic. The lower and upper bounds for its 95% confidence interval (CI) are derived from the 2.5% quantile and 97.5% quantile of the sampling distribution respectively.

3.2. A Sensitivity Analysis Method for the SIR Model

In this section, we proposed a sensitivity analysis method for the SIR model to adjust the $\beta_{t+1}(\bar{a}_t)$ under different counterfactual scenarios. Our idea is motivated by the sensitivity analysis methods proposed by Robins [9] and Brumback et.al [10]. We specified a marginal structural model [11] for $\beta_{t+1}(\bar{a}_t)$:

$$\beta_{t+1}(\bar{a}_t) = \beta_{t+1}(\bar{a}_t^{\dagger}) exp\left(\sigma \sum_{k=1}^{t} \left(a_k^{\dagger} - a_k\right)\right), \tag{S.2}$$

where σ is an unknown parameter quantifying the effect size of the hypothetical increment of vaccination coverage on the contact rate. Note that it's a model for the potential outcome. Equation (S.2) gives the relationship between the vaccination speed $\bar{a}_t$ and the potential outcome $\beta_{t+1}(\bar{a}_t)$. This model specification is common for a positive variable [11]. Note that $a_k^{\dagger}$ is observable (the vaccination speed under status quo). $\beta_{t+1}(\bar{a}_t^{\dagger})$, the contact rate under status quo, can be estimated using MLE as described in section 3.1.1.

To predict the cases under other scenarios, we

- set the parameter σ to a certain value;
- plug the MLE of $\beta_{t+1}(\bar{a}_t^{\dagger})$, the observed vaccination speed $\bar{a}_t^{\dagger}$ and the hypothetic vaccination speed under each scenario $\bar{a}_t$ into (S.2), get estimates of $\hat{\beta}_{t+1}(\bar{a}_t)$, the adjustment of $\beta_{t+1}(\bar{a}_t^{\dagger})$ is on the daily level;
- plug the estimates of $\hat{\beta}_{t+1}(\bar{a}_t)$ into SIR model (S.1), then simulate the SIR model to predict the cases under each scenario.

4. Results

The maximum likelihood estimates for $\beta^{(1)}$ to $\beta^{(6)}$ are 0.069, 0.092, 0.114, 0.104, 0.084, 0.060 respectively. The estimate for γ is 0.072.

Figure S2 to Figure S7 show the prediction by SIR models and the comparisons with SNMM by setting σ to $0, 0.05, 0.15, 0.25, 0.35, 0.45$ respectively.

In Figure S2, we choose $\sigma = 0$, which means we don't adjust for $\hat{\beta}_{t+1}(\bar{a}_t)$. In panel A of Figure S2, although the estimates of the SIR model are covered by the 95% CI of SNMM, the point estimates are relatively different, especially for the no vaccination scenario. The estimates under the 'speed up' scenario and the 'speed down' scenario are quite close.

From Figure S3 to Figure S7, we further adjust the estimates of β_t using equation (S.2) by setting $\sigma = 0.05, 0.15, 0.25, 0.35, 0.45$ respectively. We found that the prediction of the SIR model and SNMM get closer. We also found that when $\sigma = 0.35$, almost all predictions from SIR are similar to that from SNMM. In **Figure S6**, we show the results when $\sigma = 0.35$. In panel A of Figure S6, the results are pretty similar to the results shown in Figure 2 in the manuscript. For example, the comparison between the status quo and the no-vaccination scenario shows that the vaccination during the study period has effectively reduced the disease burden. We found that compared to 1% constant vaccination speed, 4% constant vaccination speed reduced more cases. Similarly, we found that compared to half vaccination speed, twice vaccination speed reduces more cases. The comparison between 'Speed up' and 'Speed down' scenarios shows the importance of accelerating vaccine rollout in the early stage. In panel B of Figure S6, we showed the similarity between the estimates from SIR models and SNMM: almost all the estimates from SIR are covered by SNMM, and the estimates from SIR are also quantitively similar to the estimates from SNMM.

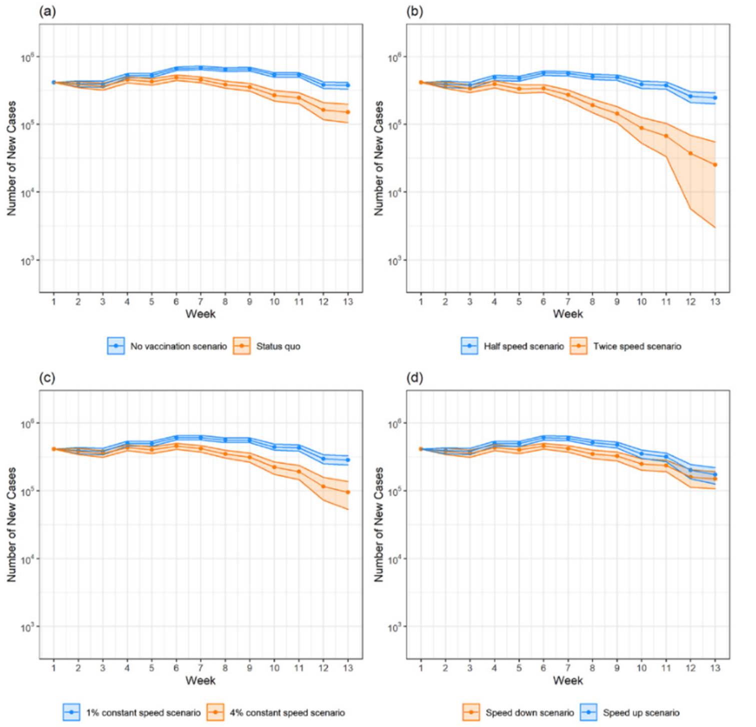

Panel A: Predicted number of new cases in each week under different scenarios using SIR model.

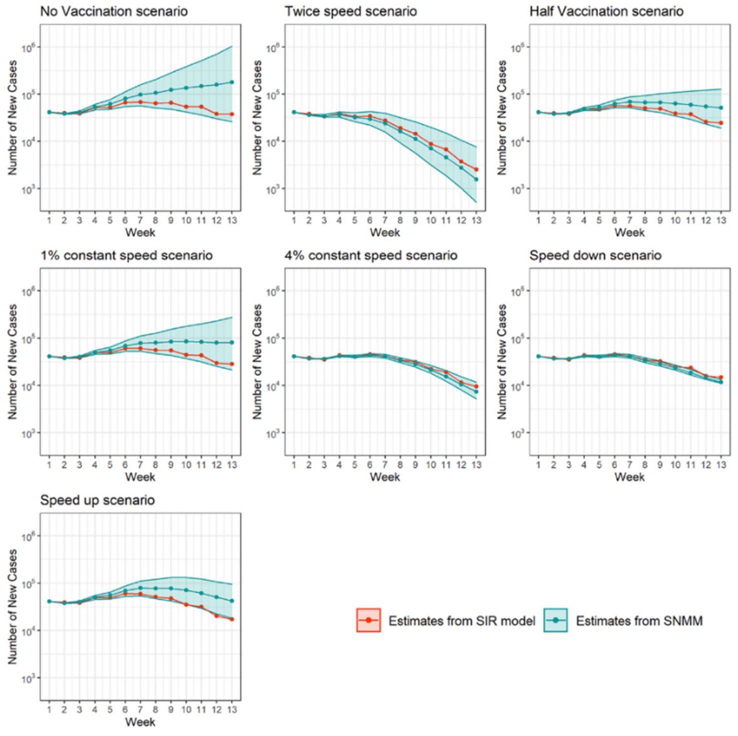

Panel B: Comparisons of predicted new cases between SIR model and SNMM.

Figure S3. Simulating the SIR model with $\sigma = 0.05$. Panel A shows the predicted number of new cases in each week under different scenarios using SIR model: (**a**) shows the comparison of predicted number of new cases under no vaccination scenario and status quo. (**b**) shows the comparison of predicted number of new cases under half speed scenario and twice speed scenario. (**c**) shows the comparison of predicted number of new cases under 1% constant speed scenario and 4% constant speed scenario. (**d**) shows the comparison of predicted number of new cases under speed up scenario and speed down scenario. Panel B shows the comparison between SIR model and SNMM. The green area is the 95% CI of SNMM.

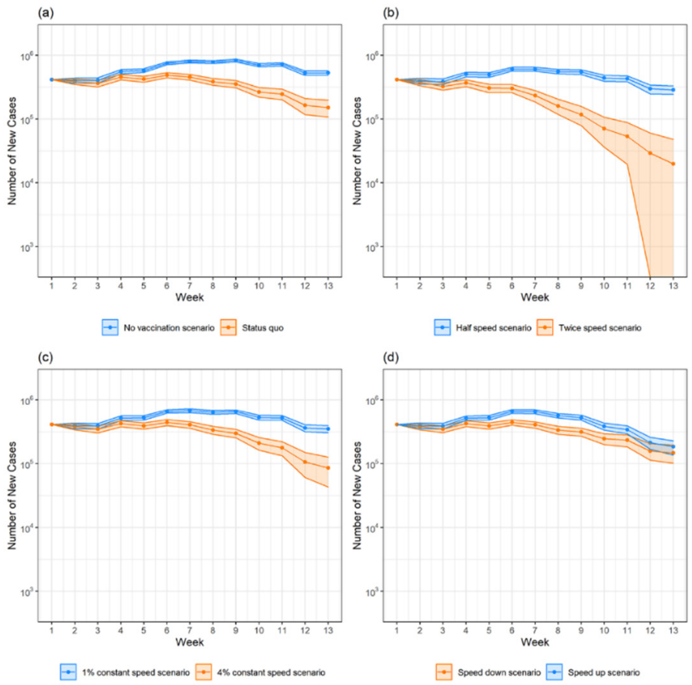

Panel A: Predicted number of new cases in each week under different scenarios using SIR model.

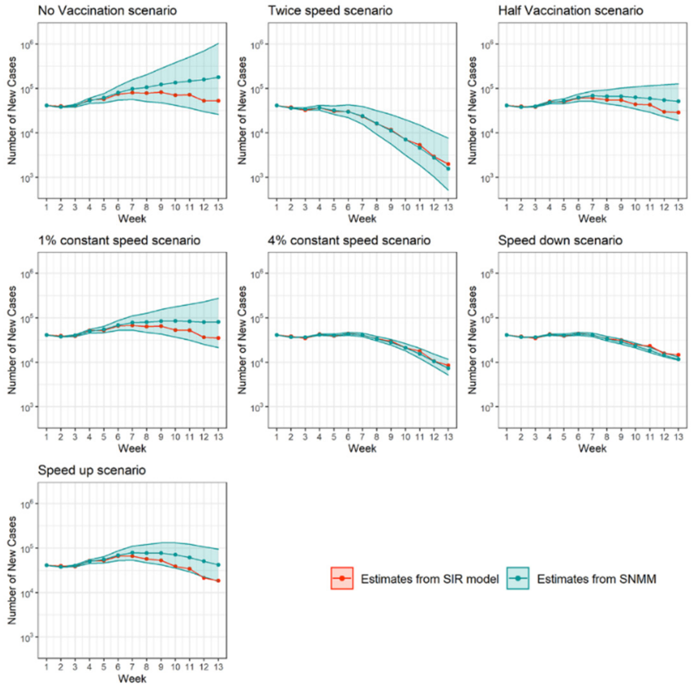

Panel B: Comparisons of predicted new cases between SIR model and SNMM.

FigureS4. Simulating the SIR model with $\sigma = 0.15$. Panel A shows the predicted number of new cases in each week under different scenarios using SIR model: (**a**) shows the comparison of predicted number of new cases under no vaccination scenario and status quo. (**b**) shows the comparison of predicted number of new cases under half speed scenario and twice speed scenario. (**c**) shows the comparison of predicted number of new cases under 1% constant speed scenario and 4% constant speed scenario. (**d**) shows the comparison of predicted number of new cases under speed up scenario and speed down scenario. Panel B shows the comparison between SIR model and SNMM. The green area is the 95% CI of SNMM.

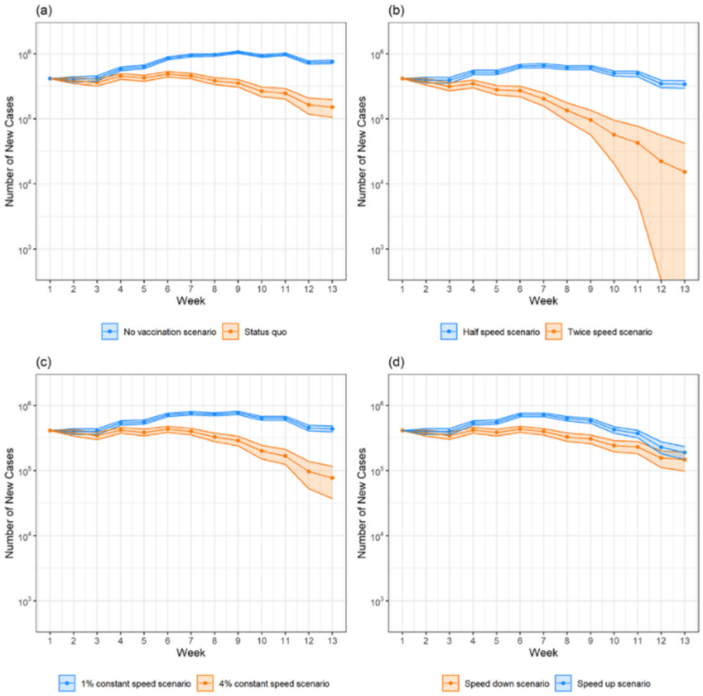

Panel A: Predicted number of new cases in each week under different scenarios using SIR model.

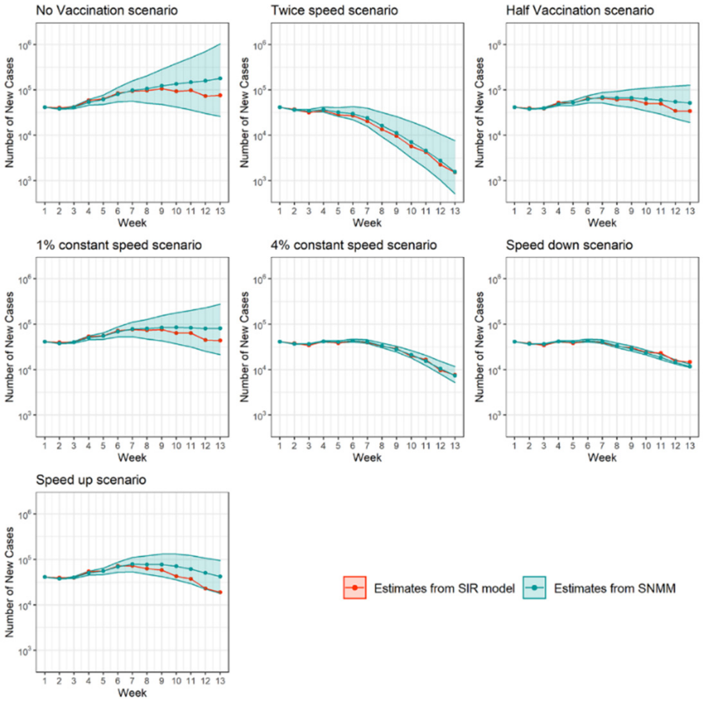

Panel B: Comparisons of predicted new cases between SIR model and SNMM.

Figure S5. Simulating the SIR model with $\sigma = 0.25$. Panel A shows the predicted number of new cases in each week under different scenarios using SIR model: (**a**) shows the comparison of predicted number of new cases under no vaccination scenario and status quo. (**b**) shows the comparison of predicted number of new cases under half speed scenario and twice speed scenario. (**c**) shows the comparison of predicted number of new cases under 1% constant speed scenario and 4% constant speed scenario. (**d**) shows the comparison of predicted number of new cases under speed up scenario and speed down scenario. Panel B shows the comparison between SIR model and SNMM. The green area is the 95% CI of SNMM.

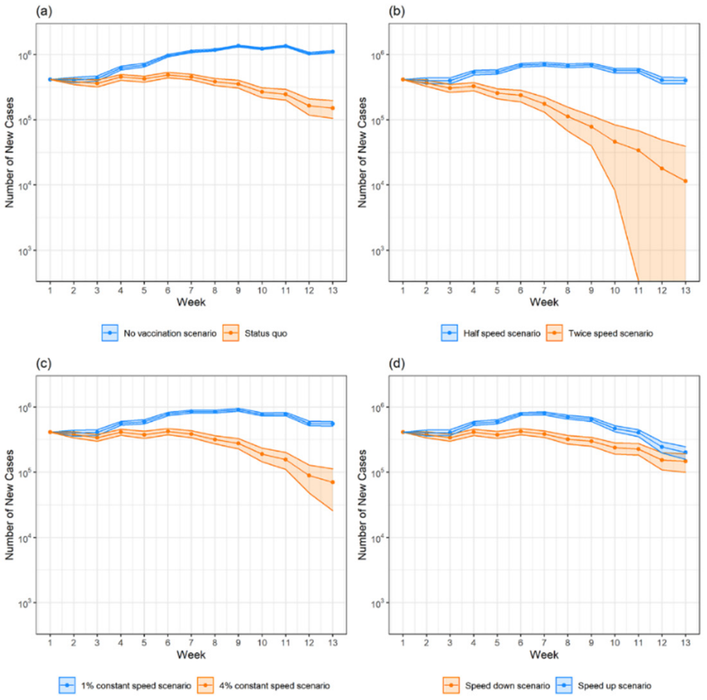

Panel A: Predicted number of new cases in each week under different scenarios using SIR model.

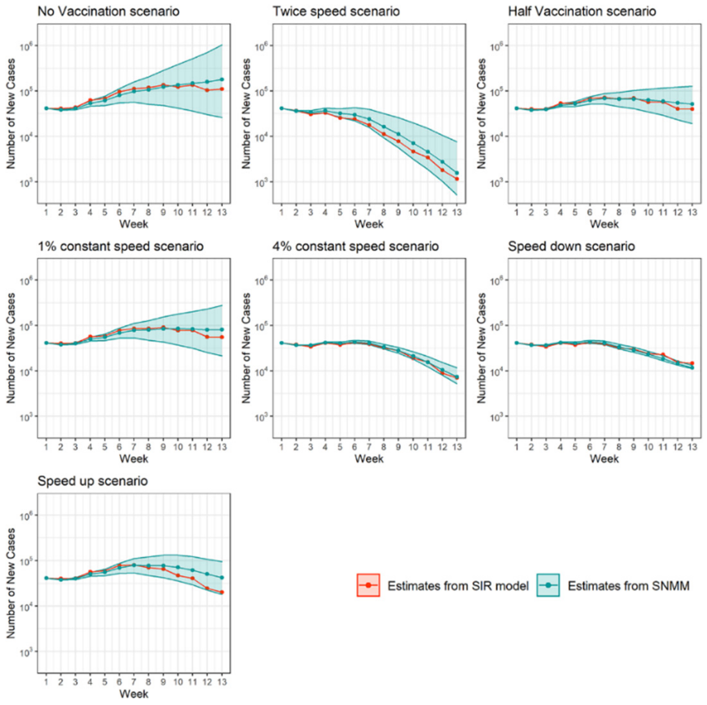

Panel B: Comparisons of predicted new cases between SIR model and SNMM.

Figure S6. Simulating the SIR model with $\sigma = 0.35$. Panel A shows the predicted number of new cases in each week under different scenarios using SIR model: (**a**) shows the comparison of predicted number of new cases under no vaccination scenario and status quo. (**b**) shows the comparison of predicted number of new cases under half speed scenario and twice speed scenario. (**c**) shows the comparison of predicted number of new cases under 1% constant speed scenario and 4% constant speed scenario. (**d**) shows the comparison of predicted number of new cases under speed up scenario and speed down scenario. Panel B shows the comparison between SIR model and SNMM. The green area is the 95% CI of SNMM.

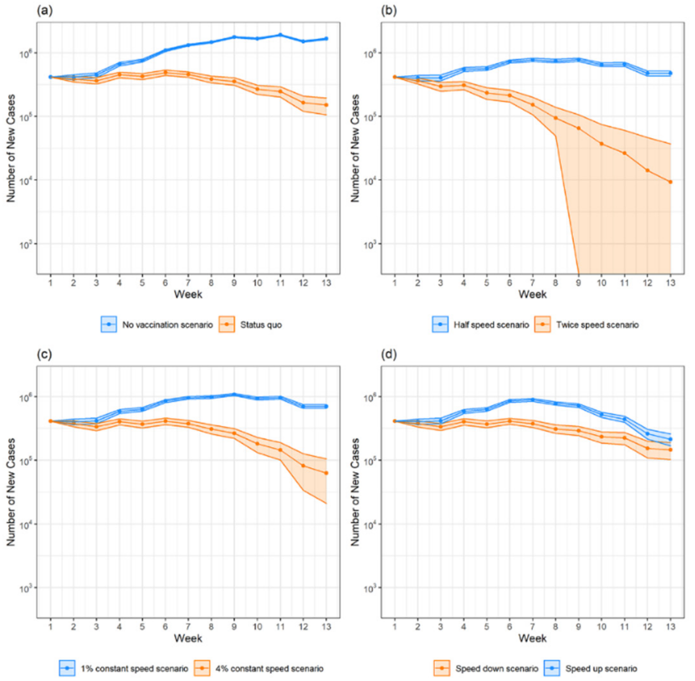

Panel A: Predicted number of new cases in each week under different scenarios using SIR model.

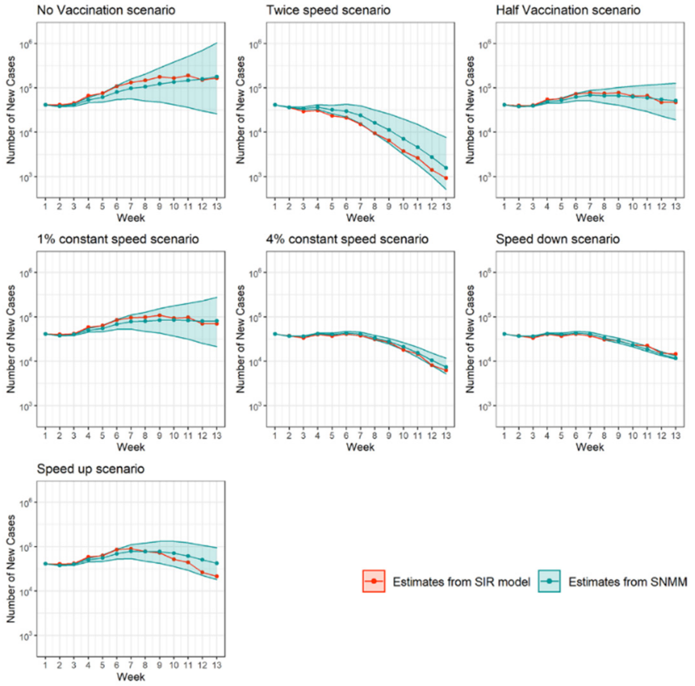

Panel B : Comparisons of predicted new cases between SIR model and SNMM.

Figure S7. Simulating the SIR model with $\sigma = 0.45$. Panel A shows the predicted number of new cases in each week under different scenarios using SIR model: (**a**) shows the comparison of predicted number of new cases under no vaccination scenario and status quo. (**b**) shows the comparison of predicted number of new cases under half speed scenario and twice speed scenario. (**c**) shows the comparison of predicted number of new cases under 1% constant speed scenario and 4% constant speed scenario. (**d**) shows the comparison of predicted number of new cases under speed up scenario and speed down scenario. Panel B shows the comparison between SIR model and SNMM. The green area is the 95% CI of SNMM.

5. Conclusions

In conclusion, the difference between the prediction from SNMM and the prediction of the SIR model may stem from the failure of adjusting time-varying confounders by the SIR model. To solve this problem, we proposed a sensitivity analysis method to adjust for β_{t+1} in the SIR model. Our sensitivity analysis shows that after adjusting for $\beta_{t+1}(\bar{a}_t)$ under model specification (S.2) and certain parameter settings, the prediction from the SIR model can be similar to the prediction from SNMM.

References

1. Glymour, M.; Pearl, J.; Jewell, N.P. Causal inference in statistics: A primer; John Wiley & Sons: 2016.
2. Bish, A.; Yardley, L.; Nicoll, A.; Michie, S. Factors associated with uptake of vaccination against pandemic influenza: a systematic review. Vaccine 2011, 29, 6472-6484, doi:https://doi.org/10.1016/j.vaccine.2011.06.107.
3. Chernozhukov, V.; Kasahara, H.; Schrimpf, P. Causal impact of masks, policies, behavior on early covid-19 pandemic in the US. Journal of econometrics 2021, 220, 23-62, doi:https://doi.org/10.1016/j.jeconom.2020.09.003.
4. COVID-19 Vaccinations in the United States,Jurisdiction. Available online: https://data.cdc.gov/Vaccinations/COVID-19-Vaccinations-in-the-United-States-Jurisdi/unsk-b7fc (accessed on 09/12/2021).
5. Bjork, J.; Inghammar, M.; Moghaddassi, M.; Rasmussen, M.; Malmqvist, U.; Kahn, F. Effectiveness of the BNT162b2 vaccine in preventing COVID-19 in the working age population-first results from a cohort study in Southern Sweden. medRxiv 2021.
6. Dong, E.; Du, H.; Gardner, L. An interactive web-based dashboard to track COVID-19 in real time. The Lancet infectious diseases 2020, 20, 533-534, doi:https://doi.org/10.1016/S1473-3099(20)30120-1.
7. United States COVID - Coronavirus Statistics - Worldometer. Available online: https://www.worldometers.info/coronavirus/country/us/ (accessed on 7 March 2022).
8. Tian, T.; Tan, J.; Luo, W.; Jiang, Y.; Chen, M.; Yang, S.; Wen, C.; Pan, W.; Wang, X. The effects of stringent and mild interventions for coronavirus pandemic. Journal of the American Statistical Association 2021, 116, 481-491, doi:https://doi.org/10.1080/01621459.2021.1897015.
9. Robins, J.M. Association, causation, and marginal structural models. Synthese 1999, 151-179.
10. Brumback, B.A.; Hernán, M.A.; Haneuse, S.J.P.A.; Robins, J.M. Sensitivity analyses for unmeasured confounding assuming a marginal structural model for repeated measures. Statistics in medicine 2004, 23, 749-767, doi:https://doi.org/10.1002/sim.1657.
11. Robins, J.M.; Hernan, M.A.; Brumback, B. Marginal structural models and causal inference in epidemiology. Epidemiology 2000, 11, 550-560.

Supplementary Material S2.

In this section, we applied the statistical methods (e.g. SNMM, GEE, fixed effects model) to estimate the effect of vaccination on weekly growth rate based on the dataset from September 2021 to December 2021 in the United States when the delta variant dominated the epidemic.

1. Methods

We collected state-level daily COVID-19 cases and vaccine coverage data from September 5 to December 4, 2021 in the US from Johns Hopkins Coronavirus Resource Center and Centers for Disease Control and Prevention (CDC) [1,2]. The baseline covariates and time-varying covariates we used were described in the main text.

Data in 44 states and Washington DC in the US were included in the analyses (We excluded Illinois, Kentucky, Michigan, New Mexico, Washington, Pennsylvania because of errors in their vaccination coverage data). The period since December 4, 2021 was excluded because the Omicron variant swiftly dominated the transmission since mid-December [3]. The period before September 5, 2021 was also excluded because the proportion of delta variant was still increasing steadily. The data were aggregated at weekly level to avoid the collection and report patterns of the confirmed cases data, resulting in the study period of 13 weeks at 45 states for analysis (first week: September 5 to September 11; the 13th week: November 28, 2021, to December 4, 2021). We estimated the effect of vaccination on growth rate of new cases using SNMM, fixed-effects models and GEE models as described in the main text.

Since the increment of vaccination coverage is about 9% from September to December 2021, we predicted the counterfactual growth rate under the following hypothetical scenarios. In each scenario, we set a different vaccination speed (denoted as $\overline{a}_{i,13}^{*}$).

- No-vaccination scenario: No people get vaccination since week 1, which means $\overline{a}_{i,13}^{*} = \bar{0}_{13}$.
- Twice speed scenario: The number of people vaccinated for the first time each week is twice of the actual number in each state, which means $\overline{a}_{i,13}^{*} = \left(2a_{i,1}^{\dagger}, 2a_{i,2}^{\dagger}, \dots, 2a_{i,13}^{\dagger}\right)$.
- Half speed scenario: The number of people vaccinated for the first time each week is half of the actual number in each state, which means $\overline{a}_{i,13}^{*} = \left(\frac{a_{i,1}^{\dagger}}{2}, \frac{a_{i,2}^{\dagger}}{2}, \dots, \frac{a_{i,13}^{\dagger}}{2}\right)$.
- 0.3% constant speed scenario: 0.3% population get their first dose in each week in each state, which means $\overline{a}_{i,13}^{*} = (30,\ 30, \dots, 30)$.
- 1% constant speed scenario: 1% population get their first dose each week in each state, which means $\overline{a}_{i,13}^{*} = (100,\ 100, \dots, 100)$.
- Speed up scenario: For the first 6 weeks, 0.3% population get their first dose in each week in each state, while for the rest 7 weeks, 1% population get their first dose in each week in each state, which means $\overline{a}_{i,13}^{*} = (30,\ 30, \dots, 30, 100, \dots, 100)$.
- Speed down scenario: For the first 7 weeks, 1% population get their first dose in each week in each state, while for the rest 6 weeks, 0.3% population get their first dose in each week in each state, which means $\overline{a}_{i,13}^{*} = (100,\ 100, \dots, 100, 30, \dots, 30)$.

2. Results

From September 5 to December 4, the vaccination coverage rate has increased from 61% to 70% in the states we included in our analysis, and 7.33 million confirmed cases were reported in the US. We plot the vaccine coverage rate and the number of new cases in each week in Figure S8.

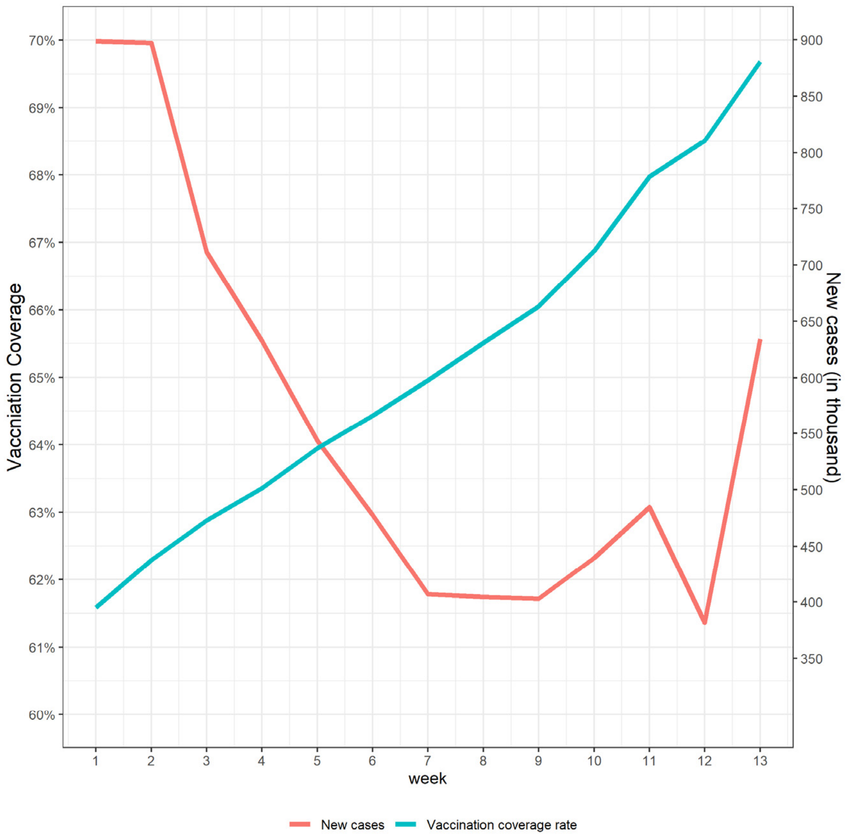

Figure S8. The vaccination coverage and new cases (in thousands) in the United States (from 5 September 2021 to 4 December 2021).

To explore the effect of vaccination on growth rate of new cases during this period, we performed the same analysis as we described in the main text and the estimated effects of vaccination on growth rate of new cases during the 13 weeks (Table S1). The estimated effects of vaccination on growth rate of new cases during the 13 weeks are shown in Table S1. The results from SNMM, GEE (adjust baseline and time-varying covariates), two-way fixed effects model and two-way fixed effects model (adjust time-varying covariates) give similar patterns. During the period from September 2021 to December 2021, the SNMM estimates the effect of 1% increase in vaccination coverage on growth rate of new cases of -0.71% (95% CI: -2.94%, 36.9%). Besides, the effect of 1% increase in vaccination coverage on growth rate of new cases is estimated as -1.59% (95% CI: -3.37%, 0.56%), -0.39% (95% CI: -2.26%, 1.47%), 0.17% (95% CI: -1.59%, 1.94%), respectively, by GEE (adjust baseline and time-varying covariates), two-way fixed effects model and two-way fixed effects model (adjust time-varying covariates). The results of scenario analysis are shown in Figure S9 and Table S.2. The sampling distribution of parameter in SNMM from bootstrap is concentrated around zero but with a right-skewed tail, which leads to the asymmetric confidence interval and wide confidence interval for estimated cases under different scenarios.

Table S1. Impact of COVD-I9 vaccine program on weekly growth rate of COVID-19 new cases.

	Effects on Growth Rate		
	Estimate	SE	95% CI
**Main analysis**			
SNMM with g-estimation	-0.71%	0.107	(-2.94%, 36.9%)
**GEE analysis**			
GEE (adjust baseline covariates)	0.99%	0.0036	(0.29%, 1.69%)
GEE (adjust baseline and time-varying covariates)	-1.59%	0.0110	(-3.37%, 0.56%)
**Fixed effect model**			
Two way fixed effect model	-0.39%	0.0095	(-2.26%, 1.47%)
Two way fixed effect model (adjust time-varying covariates)	0.17%	0.0090	(-1.59%, 1.94%)

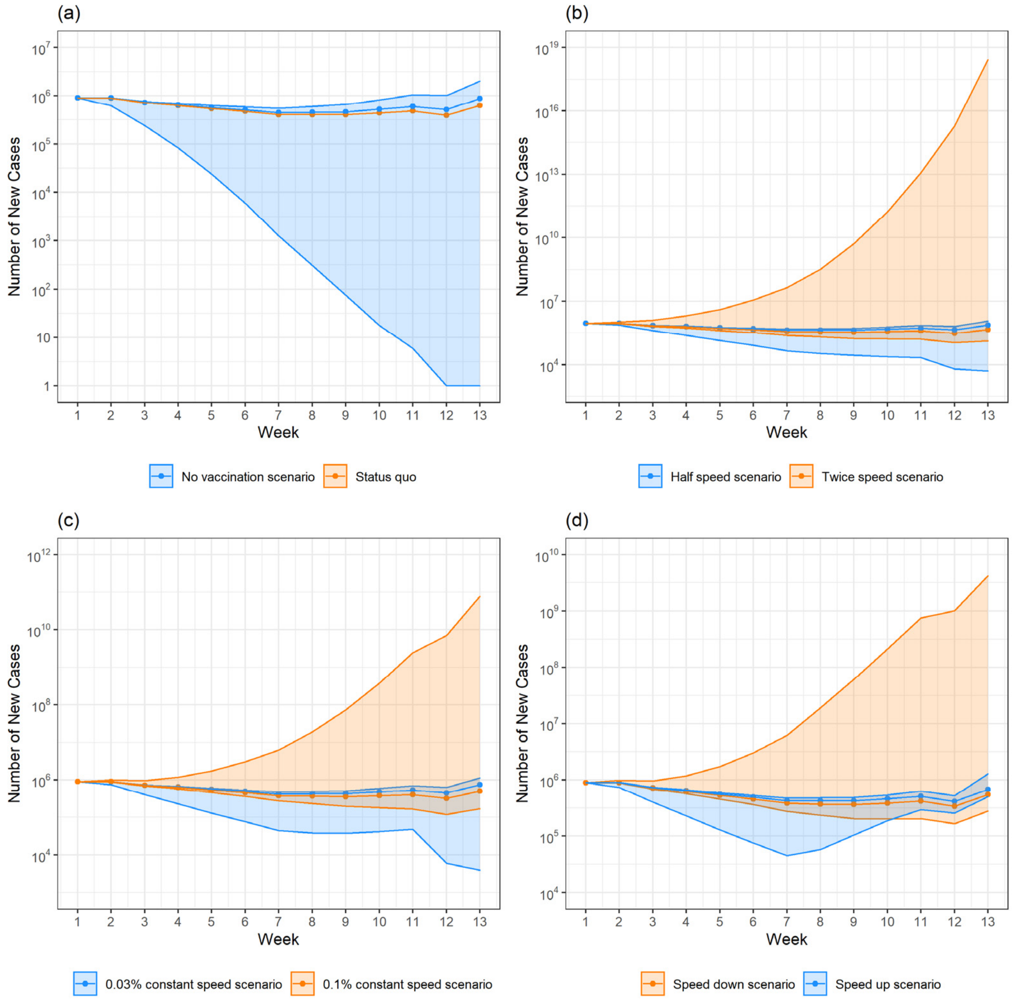

Figure S9. Predicted Number of new cases in each week under different scenarios (2021.9-2021.12): (**a**) shows the comparison of predicted number of new cases under no vaccination scenario and status quo. (**b**) shows the comparison of predicted number of new cases under half speed scenario and twice speed scenario. (**c**) shows the comparison of predicted number of new cases under 0.03% constant speed scenario and 1% constant speed scenario. (**d**) shows the comparison of predicted number of new cases under speed up scenario and speed down scenario.

Table S2. Results of the base case and scenarios analyses (2021.9-2021.12).

Scenarios	Cumulated New Cases (Million)		Vaccination Effectiveness	
	Estimate	95% CI	Estimate	95% CI
**Base case**				
Status quo	7.33	/	10.0%	(-230%, 42%)
**Scenarios analysis**				
No-Vaccination	8.14	(2.22, 12.56)	0%	/
vaccination speed: two times the status-quo speed	6.70	$(5.47,\ 1.85 \times 10^{17})$	17.7%	$(-8.35 \times 10^{16}, 56.4\%)$

vaccination speed: half of the status-quo speed	7.72	(3.14, 9.43)	5.2%	(-41.6%, 24.9%)
vaccination speed: 1% population per week	6.84	$(5.76,\ 6.24 \times 10^6)$	16.0%	$(-2.82 \times 10^6, 54\%)$
vaccination speed: 0.3% population per week	7.70	(3.11, 9.29)	5.4%	(-40.2%, 26.0%)
Speed-down: 1% for first 7 weeks and 0.3% for last 6 weeks	6.92	$(5.95,\ 1.7 \times 10^5)$	15%	$(-7.26\times 10^4, 52\%)$
Speed-up: 0.3% for first 6 weeks and 1% for last 7 weeks	7.55	(5.21, 8.85)	7.4%	(-206%, 33.1%)

3. Discussion

The estimate from SNMM is still negative but insignificant because the 95% confidence interval is very wide and 0 is included in this interval. Therefore, we can't conclude that the vaccination during the study period effectively reduced the growth rate of new cases. However, this result based on the causal model should be interpreted carefully. Note that the treatment here is the vaccination population from September 5 and December 4, which means we only found that the vaccination during the period (September 5, 2021 to December 4, 2021) didn't reduce the growth rate of new cases and this causal interpretation is only valid for the period we studied.

There may be multiple reasons for the insignificant result. First, other studies based on individual-level real-world data have shown that the efficacy of the COVID-19 vaccines against the delta strain has declined compared to that against the original strain [4]. Therefore, it is plausible that the vaccination effectiveness against the delta strain at the population level also declined compared to that against the original strain. Second, it should be noted that during this period, the increment of vaccination coverage was only 9%. Given the base vaccine coverage of about 61% before September 5, it's possible that the additional vaccination during the study period has minor effect against the delta strain. The vaccination before September 5 may have significantly impacted the transmission of the delta strain. However, it's hard to model this effect between June 2021 and September 2021 since the proportion of variants changed radically during this period. The effect of the vaccination on growth rate could depend on the proportion of the variants.

References

1. COVID-19 Vaccinations in the United States,Jurisdiction. Available online: https://data.cdc.gov/Vaccinations/COVID-19-Vaccinations-in-the-United-States-Jurisdi/unsk-b7fc (accessed on 09/12/2021).
2. Dong, E.; Du, H.; Gardner, L. An interactive web-based dashboard to track COVID-19 in real time. The Lancet infectious diseases 2020, 20, 533-534, doi:https://doi.org/10.1016/S1473-3099(20)30120-1.
3. COVID Data Tracker. https://covid.cdc.gov/covid-data-tracker/#variant-proportions. (Assessed: March-9-2022)
4. Bian, L.; Gao, Q.; Gao, F.; Wang, Q.; He, Q.; Wu, X.; Mao, Q.; Xu, M.; Liang, Z. Impact of the Delta variant on vaccine efficacy and response strategies. Expert review of vaccines 2021, 20, 1201-1209, doi:https://doi.org/10.1080/14760584.2021.1976153.
